# Optimizing engine operating parameters for enhanced performance in a combustion-enhanced ternary-fuelled compression ignition engine

**DOI:** 10.1038/s41598-025-05628-3

**Published:** 2025-07-02

**Authors:** Sinnappadass Muniyappan, Ravi Krishnaiah

**Affiliations:** School of Mechanical Engineering, Vellore Institute of Technology, Vellore, Tamil Nadu 632014 India

**Keywords:** Diesel-mahua biodiesel-ethanol blend, ZnO combustion additive, Optimal injection timing, Optimal EGR rate, ANN–RSM optimization techniques for improved engine characteristics, Energy infrastructure, Mechanical engineering, Climate sciences, Environmental sciences

## Abstract

This research aims to determine an appropriate injection timing (IT) and exhaust gas recirculation rate (EGR) for optimal output factors on a compression ignition (CI) engine fuelled by diesel-mahua-ethanol blend combined with zinc oxide (ZnO) combustion enhancer using experimentation, response surface methodology (RSM) and artificial neural networks (ANN). The generated ANN and RSM models demonstrated enhanced prediction accuracy with high correlation coefficient (R^2^) values. The effects of IT and EGR rate were experimented at varying load conditions. The RSM established operating parameters for optimal output responses are 26.4° bTDC IT and 8.63% EGR rate for B25E15Zn50 blend. Finally, the process optimization by RSM has been validated with experimental results. The established engine operating parameters resulted in improvement of peak cylinder pressure (CP), heat release rate (HRR), brake thermal efficiency (BTE) by 12.3%, 9.9%, 3.7% respectively and also reduction in hydrocarbon (HC), carbon monoxide (CO), smoke, and nitrogen oxides (NOx) by 26.4%, 19.6%, 43.6% and 33.7% respectively at 80% load. This research signifies the benefit of RSM and ANN models for establishing engine operating parameters for optimal engine output responses.

## Introduction

A country’s economic development is heavily influenced by its energy supply. Effective use of natural resources aids in satisfying energy demand^1^. Fossil fuels are rapidly being utilised in the energy industry, notably for transportation and generation. Petroleum is the most dependable and efficient energy source in both sectors^2^. The adverse effects of this dependence include the apprehension of extinction. Moreover, detrimental emissions from petroleum fuels represent a substantial risk to the environment, enhancing the greenhouse gas (GHG) impact, climate change, and the destruction of the ozone layer. It is essential to develop a novel fuel that is eco-friendly, economically viable, and reduces energy use^3^. Renewable energy is a cost-effective source of energy that is being utilised in a variety of countries as a result of innovative research and historical observations. The creative model-based production, transportation, and use of renewable energy ensures the energy, environmental, social, and financial gains. Through lower carbon footprint and control of greenhouse gas emissions, renewable energy significantly helps to slow down climate change. Furthermore, it contributes to the improvement of public health by providing a cleaner alternative to fossil fuels, which in turn reduces air pollution^4^. Many researches have indicated that biodiesel and its blends are the best alternative sources for diesel fuel, with higher performance outcomes than diesel^5^. Biodiesel is a sustainable fuel source that is also non-toxic and biodegradable^6^. An investigation on CI engine emissions and combustion parameters fuelled by B20 waste cooking oil biodiesel, resulted in HC and CO reduction, and increase in NOx and the exhaust gas temperature^7,8^. The brake thermal efficiency(BTE) and engine torque are enhanced by increasing the fuel IP^9^. The impact of carbon nanotubes (CNT) as fuel additive on CI engine performance metrics and emission resulted in reduced emissions of HCs, CO, and soot and improved BTE, power, exhaust temperature, and brake specific fuel consumption (BSFC). Diesel engines’ performance and emissions are enhanced when nanoparticles (NPs) are added to the fuel^10^. NPs facilitate combustion and transport secondary energies. A wide variety of metal oxide NPs are compatible with biofuels; those include boron, copper, iron, magnesium, silver, gold, manganese, and iron oxide. Another method to enhance fuel performance is by altering the physicochemical features of metal-based NPs, such CNT and magnesium^11^. The impact of nano-additives on the performance and emissions of a CI engine powered by diesel and biodiesel with dose of NPs influences IT, physicochemical properties, and cold flow characteristics such as viscosity, calorific value (CV), flash point, and fire point^12^. The effect of adding graphene oxide (GO) to diesel/higher alcohol mixtures on diesel engine increases BTE by 15%. On the other side, adding GO boosts NOx levels by 30% while drastically reducing CO, UHC, and smoke production levels by 40%, 50%, and 20%, respectively^13^. Deep et al. tested a single-cylinder CI engine powered by B20 (20% castor oil biodiesel and 80% diesel fuel) with varied IT and IP. Investigators modified the IT at 21°CA, 23°CA, and 25°CA, while the IP was changed at 200 bar, 250 bar, and 300 bar, respectively. It is worth noting that the BTE is slightly reduced for all IPs, as are the NOx and smoke levels^14^. Venu et al. examined the impacts of modifications in diesel engines with varying IP, IT, combustion chamber geometry, etc. in order to determine the optimal operating condition. High IPs with advancement/retardation of IT can significantly enhance engine performance with lower HC, CO, NOx, and smoke by improving combustion and air–fuel mixing rate^15^. Diesel engines can be fuelled with a variety of biodiesel mixes to minimise pollutants. The addition of NPs to biodiesel blends further lowered GHG such as CO, carbon di-oxide (CO_2_), HC, and NOx. Furthermore, by optimising the IP, IT, and loading conditions, the engine produces less emissions. Engine testing were conducted by Kumar et al. using Pongamia B20 biodiesel and ferrofluid added. Ferrofluids are water-based suspensions of NPs based on ferrous materials. The ferrofluid solutions containing B20 biodiesel were tested at concentrations of 0.5, 1.0, and 1.5%. When comparing a 1% ferrofluid solution to a plain B20, brake specific energy consumption (BSEC) decreased by 8%. Similarly, the 1% ferrofluid solution with B20 reduced CO by 35.8% and HC by 22.9% when compared to B20 blend^16^. Hasannuddin et al. performed research on nano-additive doped water in diesel emulsion fuel to improve performance parameters. Aluminium oxide (Al_2_O_3_), copper oxide, magnesium oxide, and ZnO nano additions were used for doping with 10% water in a diesel emulsion. The concentration of NPs was consistently maintained at 50 ppm across all testing. The Al_2_O_3_ addition exhibited the greatest NOx and the lowest CO and BSFC compared to other additives^17^. Furthermore, different fuel IT and IP strategies are employed to improve engine performance. By improving early combustion and raising cylinder temperature, advanced fuel injection reduces CO and HC. While BTE and NOx are impacted by combustion chamber pressure, late fuel injection reduces the combustion duration (CD). Experiments on the properties of CI engines with different input parameters were time-consuming and expensive. Recently, the issue has been possible to predict the data set and optimise the input variables of the internal combustion (ICE) engine^18^. Melvin et al., examined the effective method to reduce pollutants and improve the combustion characteristics of hexanol diesel blend fuel by investigating on varying IT and EGR. 30% Hexanol at 25°CA IT and 30% EGR has shown to be a useful combination that resulted in a 35.9% reduction in smoke and a 3% increase in NOx^19^. The findings of using the EGR system showed that the peak pressure was significantly lowered and the peak pressure point was shifted away from TDC by the impact of the EGR. Additionally, it was concluded that at lower EGR rate of 0–30%, minimal variation was seen in soot and NOx^20^. Ashok et al. found that EGR reduced NOx without affecting engine efficiency. NOx dropped 51% with 10% EGR compared to 0%. As a trade-off, decreasing NOx using EGR affects engine performance. Many studies investigated NPs for combustion improvement to face such trade-off behaviour. NPs have increased oxygen (O_2_) content, surface to volume(S/V) ratio, and catalytic activity^21^. A study found that metal-oxide NPs enhance biodiesel engine performance at all dosages because of micro-explosion feature greatly increasing fuel evaporation and vaporisation^22^. Haozhong et al. focused on employing EGR technology to reduce soot and NOx using a dual fuel combination of n-pentanol and diesel. Blends of fuels have their cetane number (CN) increased by adding ethyl hexyl nitrate(EHN), a cetane enhancer. Both the 20% and 40% EGR fuel evaluations were conducted in a turbocharged CI engine. The addition of EHN to pentanol resulted in a reduction in soot particles as well as a significant drop in HC and CO. Further implementation of the 40% EGR resulted in an increase in HC levels to 1.37 g/kWh and CO levels to 9.11 g/kWh. Different post-treatment technologies like selective catalytic reactor, adjusting IT and fuel IP, modifying combustion chamber morphologies, differential geometry turbocharging, and NP mixing all help to lower engine emissions and increase BTE^23^. Due to the resources, manpower, and machine hours needed for fuel production, repeated research studies to evaluate the impacts of NP on biodiesels may be costly. Nevertheless, the use of contemporary statistical-mathematical methods and improved computing capacities may help to preserve these finite resources and lower the expenses of experimental study^24^. To accurately forecast the operating characteristics, the RSM model was developed. The same quantity of surfactant and dispersant had to be added in order to change the chromium tri-oxide (Cr_2_O_3_) NP concentration to 60, 80, and 100 ppm. In comparison to the other fuels, BD20 + Cr_2_O_3_ 80 mg/L + DSP 80 mg/L increased BTE by 16.58% and decreased BSFC by 0.58%. CO dropped 31.85% at maximum load at compression ratio (CR) 18.5:1; NOx dropped by 6.16%; HC down by 22.23%; and smoke by 62.61%. For every output parameter, the R^2^ for ANN and RMS methods ranged from 0.96 to 0.98^25^. In a study three input layers, 25 hidden levels, and 8 output layers made up the an ANN model. With an correlation co-efficient (R^2^) near to 1, the findings showed that the experimental data sets and the model predictions were in excellent agreement^26^. Several software programs have been deployed to improve the fuel map planning process. Many of these computer programs got multiple simulations with high accuracy with limited the number of tests. Taguchi software calculate the ideal quantities of all variables and identified most significant variable. RSM and ANN are used to assess the impact of each input variable on the output variable. A hybrid technique can enhance process efficiency by combining ANN model-based prediction with RSM to identify the best combination of two or more input variables^27,28^. Using ANN and RSM techniques, Krishna et al. investigated which engine parameters were most effective in reducing emissions while maximising performance. At varying CRs and engine loads, the trials were conducted on diesel engines that use mixtures of diesel, plain vegetable oil, and diethyl ether as fuel. An error rate of less than 5% was observed for both ANN and RSM. Consequently, they concluded that ANN and RSM were crucial in improving engine efficiency and cutting down on pollution^29^. Given the limitations affecting the predictive ability of RSM, it is anticipated that the progression of artificial intelligence (AI) algorithms and the ongoing development of material databases will substantially contribute to improving the accuracy of predictions made by models such as ANN^28^.

The combined impact of the IT and the EGR was designed with the goal of achieving low NOx levels while maintaining efficiency. According to the extensive research review, the occurrence of trade-off effects on performance and emissions can be efficiently optimised by evaluating the engine design parameter, control parameter, and fuel reformulation strategy simultaneously. The current work proposes a multi-response optimisation approach for determining the most desirable combination of fuel IT and EGR rates. The objective is to maximise performance while minimising emissions from the diesel engine with acceptable precision. This study used different EGR rates (5%, 10%, and 15%) and injection timings (21°, 23°, and 25° bTDC) to minimize NOx and smoke while improving performance. Based on our prior research, the D60B25E15 fuel mix with 50 mg/l ZnO combustion enhancer was chosen for additional investigation due to its excellent performance, emissions, and combustion characteristics. This study examined the influence of catalytic combustion activity of the fuel blend and ZnO at both advanced and retarded IT and EGR rates considering engine output responses viz, peak CP, HRR, BTE, BSEC, and emissions such as HC, CO, NOx, and smoke, all of which contribute to effectual engine performance and lowered environmental damage. To accurately and effectively find a solution for the research problem, this study employs RSM and ANN models using minimal experimental data. Models were first trained for prediction and optimisation. RSM and ANN models optimise and forecast different engine parameters in order to achieve maximum output while minimising emissions. These optimisation methods are used to create a predicting optimised model.

## Materials and methods

### Biofuel production process

Free fatty acids (FFA) make about 21% of the inedible raw oil from Mahua. Mahua methyl ester (MME) was created using a two-step transesterification process in order to reduce the FFA to 1%. An acid-catalyzed procedure was used to complete the first stage. 500 ml of heated preheated Mahua raw oil was added to a mixture containing 0.35 v/v, of 175 ml of methanol, and 1% v/v, of 5 ml of concentrated sulfuric acid. For one hour at 60 °C, the whole 680 ml solution container is set on a mantle heater and constantly stirred at 500 rpm. The product was used for base transesterification after the acid transesterification procedure. The second phase was carried out utilising an alkaline catalyst technique, which included adding 0.25 v/v, or 125 ml, of methanol and 0.7% w/v, or 3.5 gm, potassium hydroxide, as catalyst to a product that was created during the acid transesterification reaction. To create biodiesel, the entire solution container is placed on a mantle heater and constantly stirred at 900 rpm for one hour at 60 °C. The collected biodiesel from the second phase of transesterification was mixed with warm distilled water at 50 °C and agitated rapidly. After a few minutes, the water was allowed to drain down the bottom of the separating funnel. Until a clear biodiesel was produced, this process was repeated 3 times. After adding anhydrous calcium chloride (CaCl_2_) to the biodiesel, it was gradually heated to 50 °C. To create a clean Mahua methyl ester biodiesel, the biodiesel containing anhydrous CaCl_2_ was agitated forcefully and then removed from the biodiesel. At last, the production of MME biodiesel reaches 85%^30^.

### Fuel blend preparation

The mixture included 15% ethanol, 25% mahua biodiesel, and 60% diesel. Biodiesel acts as a co-solvent and bonding agent between diesel and ethanol fuels. Tests have been conducted to assure the physical and thermal stability of nano additives by adding various kinds of nano additives to fuel^31^. In this work, the NPs used in the fuel blend ranged in size from 20 to 30 nm. ZnO NP was chosen to combine with B25 blend, ethanol, and diesel to make the nano blend (B25E15). The optimal ZnO NP concentration of 50 mg/L (50 ppm) was included into the fuel mixture using an ultrasonic mixer. An ultrasonic mixer and magnetic stirrer were utilised for 30 min to prevent the development of agglomerated and aggregated NPs, as well as to reduce agglomerated NPs to the nanometre size. To avoid sedimentation or buildup during preparation, the nano mixture was stirred for 30 min more using an ultrasonic mixer. To test the stability of nano blends, samples were kept and stored under glass for 21 days. During a one-month experiment, the nano mixes were found to be stable and suitable for blending. In this work, surfactants were added to the nano blend to limit the possibility of agglomeration during the preparation process^32^.

### RSM optimization techniques

The RSM uses design expert software to create the input parameters and analyse engine performance. To assess the greatest range of variance with the lowest number of tests, the design of experiment(DoE) idea is offered. The regression equation is used in regression analysis (Sect. “Regression equation in engine parameters”) to forecast the untested value response. subsequently saves time and effort to use the statistically efficient model to predict the response of any non-experimental input. Design Expert 13 was used to design the RSM model. In order to optimise the process parameters and investigate the response surface, the design matrix contains experimental trial runs that different combinations of input variables. The first step involves choosing the parameters that will be used for both input and output. The central composite design (CCD) was used in this investigation with the factors set at various levels for all variables. This research designated engine output characteristics as responses, whereas varying injection strategies, EGR concentrations, and engine loads were identified as input factors. This design matrix and optimisation employing CCD in RSM methods made use of a variety of process parameters. During the experiment, the following responses were recorded: peak CP, HRR, BTE, BSEC, CO, HC, NOx, and smoke. A wide range of input parameters was found by subtracting the bottom and upper limits, which were based on a thorough literature research. These findings provide information on how various process factors influence the engine characteristics^33^. Analysis of variance (ANOVA) was used to identify significant values between input variables and responses as shown in Table 1. To determine whether the proposed model is compatible with test results, use statistical parameters such as regression co-efficient (R^2^), Adj. R^2^ as shown in Table 2, F-test value (F-value), and probability value (p-value). The higher the F-value and the lower the P-value, the more relevant the associated term in the proposed correlation for response; hence, a P value less than 0.05 is regarded significant. To assess the suggested model’s compatibility with experimental data, it is essential to analyse the test outcomes using statistical measures such as R^2^, adjusted R^2^, F-value, and P-value. The decreased P value and elevated F value indicate a robust significance of response correlation. A P-value below 0.05 was deemed significant. Furthermore, the independent variables explain 99.8% of the variability in engine responses, indicating that the model does not simply explain 0.2% of the variance. Predicted R^2^ is a measure of how well the model predicts a given response value. To be in fair agreement, the adjusted R^2^ and anticipated R^2^ must be within 0.20 of one another. If they are not, there might be an issue with the data or model. In our example, the projected R^2^ of 0.9980 is consistent with the modified R^2^ of 0.9431. Adequate accuracy assesses the each responses and compares the anticipated values at the design points to the average prediction error. A significance test for the regression model and individual coefficients, assessing model adequacy, was conducted to ensure proper model fitting. Typically, the principal components were prioritised according to the F-value or P-value (probability value) at a 95% confidence level. The model’s accuracy has been assessed using the R^2^ and other parameters obtained from the ANOVA. The models and the variables’ statistical significance have been evaluated at a probability level (P < 0.05). This implies that the experimental outcomes and the model’s predicted outcomes are highly correlated. The produced model works well with the real engine out response, as shown by the low P-value and high R^2^. The engine operating variables are optimised in this project using the RSM optimiser tool, which is utilised to optimise multiple objectives. Maintaining a high BTE while reducing engine emissions of HC, CO, NOx, and smoke is the main objective of the optimisation process. Table 3 and 4 represents the parameters input level and experimental matrix^34^.

**Table 1:** ANOVA results for various engine parameters.

Source	F-value	p-value	F-value	p-value	F-value	p-value	F-value	p-value	F-value	p-value
Parameter	Peak CP		HRR		BTE		BSEC		CO	
Model	46.46	0.0001	153.68	< 0.0001	8787.35	< 0.0001	2803.25	< 0.0001	131.01	< 0.0001
A-EGR rate (%)	1.92	0.2155	15.71	0.0074	2661.57	< 0.0001	996.27	< 0.0001	10.73	0.0169
B-IT (° CA)	10.17	0.0189	342.44	< 0.0001	26,623.20	< 0.0001	10,519.50	< 0.0001	516.22	< 0.0001
AB	0.8284	0.0378	0.0083	0.0304	297.24	< 0.0001	433.33	< 0.0001	18.64	0.0050
A^2^	218.42	< 0.0001	383.74	< 0.0001	12,444.77	< 0.0001	2006.70	< 0.0001	105.78	< 0.0001
B^2^	0.9637	0.0342	26.47	0.0021	1909.95	< 0.0001	60.46	0.0002	3.67	0.0039

Source	F-value	p-value	F-value	p-value	F-value	p-value				
Parameter	HC		Smoke		NOx					
Model	768.67	< 0.0001	23.04	0.0008	53.53	< 0.0001				
A-EGR rate (%)	1171.20	< 0.0001	4.95	0.0677	139.65	< 0.0001				
B-IT (° CA)	880.08	< 0.0001	35.46	0.0010	77.98	0.0001				
AB	32.86	0.0012	1.49	0.2677	23.32	0.0029				
A^2^	1665.70	< 0.0001	65.61	0.0002	0.5760	0.0466				
B^2^	93.49	< 0.0001	7.69	0.0323	26.13	0.0022

**Table 2:** Model evaluation.

ANOVA	Peak	HRR	BTE	BSEC	CO	HC	Smoke	NOx
Std. dev	0.3688	0.4966	0.1287	0.2136	0.0015	0.3898	0.4117	5.50
Mean	73.31	68.48	30.46	12.33	0.2090	72.02	10.63	84.53
R^2^	0.9994	0.9984	0.9930	0.9854	0.9999	0.9998	0.9960	0.9999
Adjusted R^2^	0.9932	0.9829	0.9926	0.8399	0.9989	0.9979	0.9565	0.9985

**Table 3:** Engine input level.

Engine input	Code	Level		
		− 1	0	1
EGR rate (%)	A	0	10	15
IT (° CA bTDC)	B	21	23	25

**Table 4:** RSM Metrics design.

Run	A: EGR rate (%)	B: IT (° CA bTDC)
1	0	21
2	0	23
3	0	25
4	5	21
5	5	23
6	5	25
7	10	21
8	10	23
9	10	25
10	15	21
11	15	23
12	15	25

### Regression equation in engine parameters

1 $${\text{Peak}}\;{\text{CP}}\left( {{\text{bar}}} \right) = {78}.{132} + 0.{8}0{8} \times {\text{A}} + {1}.0{73} \times {\text{B}} - 0.{4}0{9} \times {\text{AB}} - {7}.{995} \times {\text{A}}^{{2}} - 0.{573} \times {\text{B}}^{{2}}$$

2 $${\text{HRR}}\left( {{\text{J}}/^\circ {\text{CA}}} \right) = {72}.{455} - 0.{737} \times {\text{A}} + {2}.{715} \times {\text{B}} - 0.{282} \times {\text{AB}} - {5}.{278} \times {\text{A}}^{{2}} - {1}.{558} \times {\text{B}}^{{2}}$$

3 $${\text{BTE}}\left( \% \right) = {31}.{71}0 - 0.{425} \times {\text{A}} + {1}.{239} \times {\text{B}} - 0.{179} \times {\text{AB}} - {1}.{571} \times {\text{A}}^{{2}} - 0.{566} \times {\text{B}}^{{2}}$$

4 $${\text{BSEC}}\left( {{\text{MJ}}/{\text{kWh}}} \right) = {12}.{157} + 0.{161} \times {\text{A}} - 0.{483} \times {\text{B}} - 0.{135} \times {\text{AB}} + 0.{388} \times {\text{A}}^{{2}} - 0.0{63} \times {\text{B}}^{{2}}$$

5 $${\text{CO}}\left( \% \right) = 0.{196} + 0.00{4} \times {\text{A}} - 0.0{43} \times {\text{B}} - 0.0{14} \times {\text{AB}} + 0.0{33} \times {\text{A}}^{{2}} - 0.00{7} \times {\text{B}}^{{2}}$$

6 $${\text{HC}}\left( {{\text{ppm}}} \right) = {64}.0{57} + {5}.{581} \times {\text{A}} - {4}.{494} \times {\text{B}} - {1}.{166} \times {\text{AB}} + {1}0.{986} \times {\text{A}}^{{2}} + {2}.{786} \times {\text{B}}^{{2}}$$

7 $${\text{Smoke}}\left( {\text{N}} \right) = {8}.{385} - 0.{577} \times {\text{A}} - {1}.{164} \times {\text{B}} - 0.{47}0 \times {\text{AB}} + {2}.{9}0{8} \times {\text{A}}^{{2}} + 0.{939} \times {\text{B}}^{{2}}$$

8 $${\text{Nox}}\left( {{\text{ppm}}} \right) = {91}0.{319} - {13}0.{22} \times {\text{A}} + {88}.{831} \times {\text{B}} + {65}.{179} \times {\text{AB}} - {14}.0{25} \times {\text{A}}^{{2}} - {89}.0{66} \times {\text{B}}^{{2}}$$

### ANN optimization techniques

Widely used artificial intelligence technology called ANN is a great tool for estimating various engine running factors for diesel and petrol engines. Especially in fields where traditional and numerical techniques are insufficient, ANN can solve a broad range of technological and scientific challenges. Its capacity to learn about the system that may be replicated without previous knowledge of transactional interactions distinguishes from the usual modelling tools. ANN creates an analytic model to tackle problems in estimating and decision-making techniques. An ANN’s estimate is often faster than conventional simulation software or mathematical models since there are no extensive iterative computations necessary to resolve differential equations using numerical approaches. The choice of a suitable network is essential for model accuracy^35^. The backpropagation approach is among the most prevalent ways in this field, despite the existence of other alternative tactics aimed at enhancing the estimation accuracy of ANN models. It typically has a minimum of three layers: an input layer, a hidden layer, and an output layer. The quantity of nodes within each stratum can vary. The input and output layers need data from experimental sources to generate the system simulation. After choosing a network design and building hidden layers, training is done until the network recognises patterns in the input. After validation, the final ANN model is authorised for use in prediction. An ANN’s learning method depends on identifying data differences and adjusting intelligence via backpropagation in order to achieve the desired outcome. As a result, deep learning is seen to be a more accurate option than other modelling techniques. The ANN implementation study models estimating performance is assessed using mean square error (MSE) and mean relative error (MRE). During the learning stage, the MSE is referred to as the error diagnostic. To evaluate the efficiency of the network, one uses the R^2^ and the MRE. The ‘R^2^’ value ranges from − 1 to + 1. Better outcomes are also possible when the value is near to + 1. The ANN produces high-accuracy results when modelling the output parameters. Nearly all data are scattered over the 45° line, indicating great agreement between test results and ANN predicted outcomes. The R^2^ values between test and expected outputs indicate that the ANN model trained with test data accurately estimated BTE and exhaust emissions (CO, HC, smoke, and NOx) using the optimum operating blend. This research used a neural network to predict diesel engine parameters using an optimized mixture of B25E15Zn50 blend. Output variables were chosen based on peak CP, HRR, BTE, BSEC, CO, HC, smoke, and NOx, whereas input parameters were different IT and various EGR rates. In this research, the neural network approach was also used to the same data set, which consisted of 11 test trials recognised by the CCD. Specifically, 14 examples (or 70% of the data trials) were used for neural network training, 3 cases (15%) for verification, and 3 cases (15%) for testing. The ANN was constructed using a feed-forward backpropagation network type, a maximum-likelihood performance function, and a training function. In order to illustrate complex issues in system modelling and identification, the feed-forward backpropagation network type was used^36^. According to the findings, the two-neuron network has the optimum combination of low MSE Eq. (9), low MRE in Eq. (10), and high R^2^ Eq. (11) engine properties.

9 $${\text{MSE}} = \frac{1}{n}\mathop \sum \limits_{i = 1}^{n} \left( {X_{exp} - X_{ANN} } \right)^{2}$$

10 $${\text{MRE}} = \frac{1}{n}\mathop \sum \limits_{i = 1}^{n } \frac{{\left| {X_{exp} - X_{ANN} } \right|}}{{\left( {X_{exp} } \right)}}$$

11 $${\text{R}}^{{2}} = {1} - \left[ {\frac{{\mathop \sum \nolimits_{i = 1}^{n} \left( {X_{exp} - X_{ANN} } \right)^{2} }}{{\mathop \sum \nolimits_{i = 1}^{n} \left( {X_{exp} } \right)^{2} }}} \right]$$

The first step in evaluating the accuracy of neural network identification is to predict the network’s output from the input data that was obtained. Specifically, 14 distinct ANN models were employed to predict the dependent variables of engine characteristics. Table 5 displays the results of the neurone independent study. Results show that the 11-neuron has the best R^2^, lowest MSE, and lowest MRE. This model is absolutely relevant to this research, but it allows RSM or other optimisation methods to verify experimental optimisation outcomes. This work should support ANN as an accepted technique for engine optimisation, according to the author. This is a promising indicator for using ANN to predict engine responses. This helps decrease the cost and time of further experimental testing by obtaining non-experimental values using ANN prediction and RSM optimisation. The RSM and ANN outcomes were confirmed and verified with the experimental findings in with each other.

**Table 5:** ANN optimal engine characteristics.

ANOVA	Peak CP (bar)	HRR (kJ)	BTE (%)	BSEC (MJ/kWh)	CO (%)	HC (ppm)	Smoke (N)	NOx (ppm)
MSE	4.035	34.892	3.560	7.039	32.801	2.084	17.590	3.780
MRE	0.078	1.654	0.098	0.137	1.226	0.005	2.790	0.038
R^2^	0.983	0.973	0.987	0.976	0.989	0.994	0.998	0.985

## Experimental set-up

**Fig. 1:**
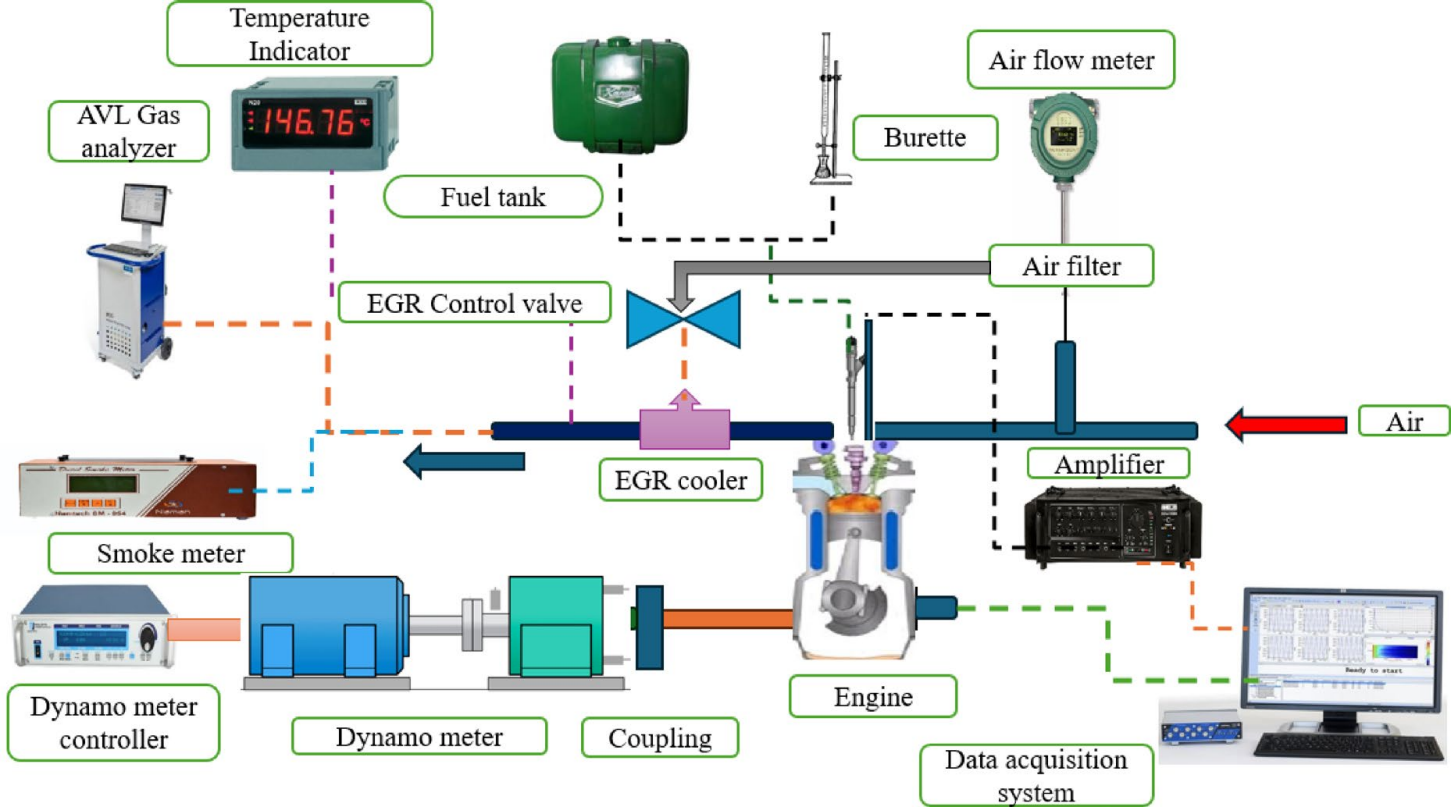
Engine test layout.

Experiments were carried out in a single cylinder 4-stroke water-cooled direct injection CI diesel engine utilising an eddy current dynamometer with EGR set-up as shown in Fig. 1. These tests were performed at different engine loads while maintaining a constant speed of 1500 rpm, with an IP of 220 bar and a CR of 17.1. The AVL gas analyser and smoke meter were installed in the tailpipe end for emissions analysis. The engine load was measured using a load cell. Various load intervals, namely 20%, 40%, 60%, 80%, and 100%, were evaluated. The engine was first warmed up by conducting tests with diesel for 20 min. The experimental trials were conducted using a B25E15 blend containing 50 mg/l of ZnO NP without EGR. B25E15 blend is used to function at varied engine IT without EGR conditions to assess NO and smoke.

Engine maps were written and updated using the linked computer for the electronic control unit (ECU). EGR levels, boost pressure, and injection strategy (timing and pressure) were all modified. A readback facility was provided for compensating varying engine conditions (temperatures, flow rates) and injection techniques (pressure, timing). Injection pressure is maintained constant by a high-pressure fuel pump and injection control unit. For varying load conditions, the ECU of the common-rail system regulate the injection timing. Subsequently, engine modifications were implemented to efficiently diminish NO and smoke by introducing EGR. Additional decrease in NO and smoke achieved with the use of the EGR technique at varying rates of 5%, 10%, and 15%, using various IT approach. An externally cooled EGR system was employed to lower the charge temperature while increasing the density of diluent gases (CO_2_ and H_2_O). Tap water was used as the cooling medium in the EGR cooler. Exhaust gases cooled to 35 °C, and EGR control valves controlled the mass flow rate into the mixing zone. The EGR valve controlled the EGR rate. The orifice monitors the flow of exhaust gases. The incoming air and mixing rate accelerated the recirculation of exhaust gas to the input manifold.

### Uncertainty analysis

Errors and uncertainties may be caused by a variety of reasons, including instrument selection and calibration, fluctuations in ambient conditions, and testing and observation procedures, among others. Uncertainty is often classified into two types: fixed mistakes and random errors. The first scenario concerns repeatability, whereas the second concentrates on analytical measures. Table 6 represents the errors value of each parameters.

**Table 6:** Accuracy and uncertainty of equipment’s.

Instrument	Range	Accuracy	% Uncertainty
Eddy current dynamometer	0–3.5 kW	± 1 kW	0.9
Fuel flow meter	0–100 ml	± 1%	0.1
Piezoelectric transducer	0–200 bar	± 5%	1
Smoke meter	0–100 HSU	± 0.1%	1
CO	0–10%	± 5%	0.03
HC	0–20,000 ppm	± 5%	0.5
NOx	0–5000 ppm	± 10%	10

## Results and discussion

### Combustion analysis

#### In-cylinder pressure

**Fig. 2:**
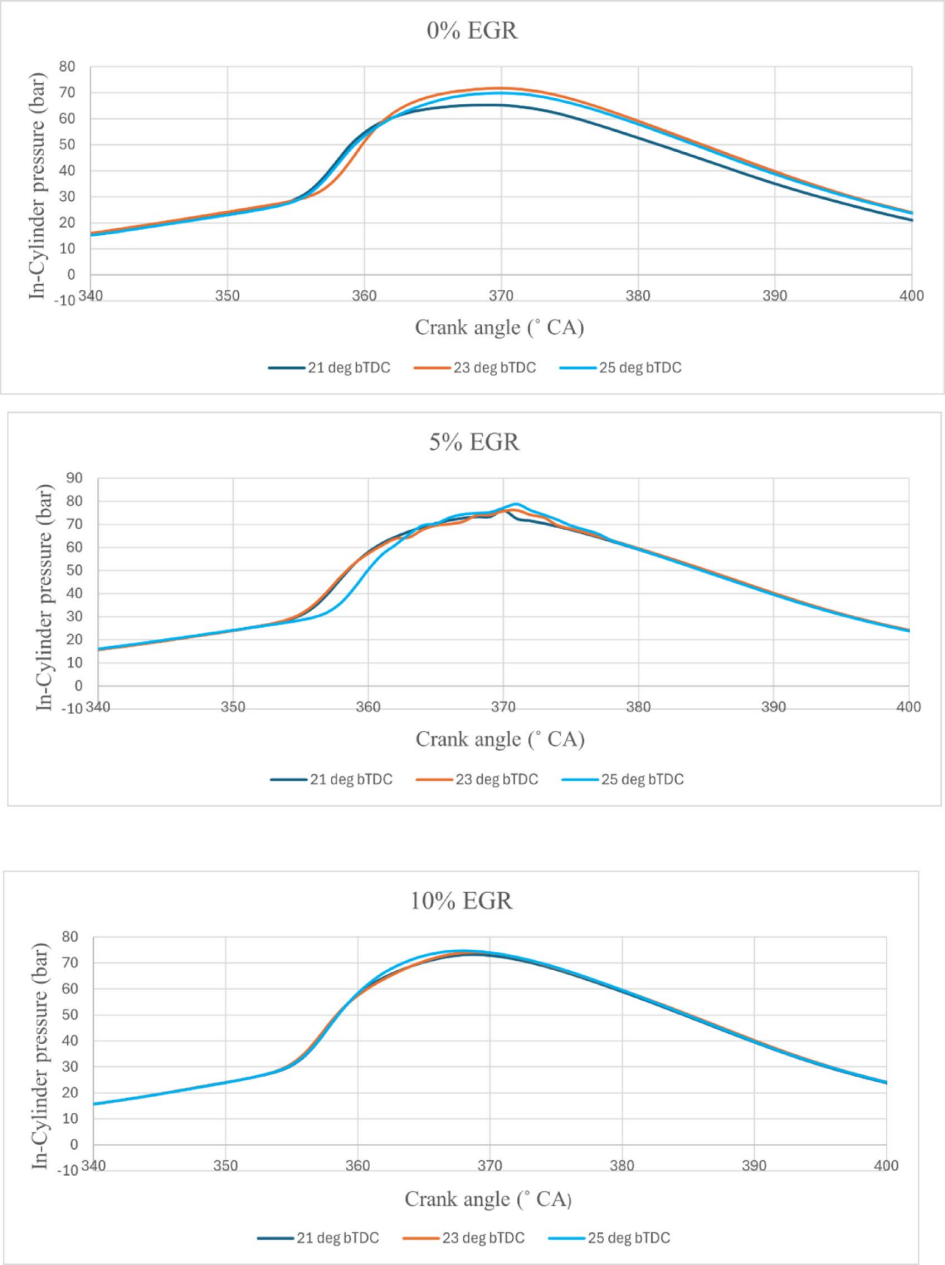

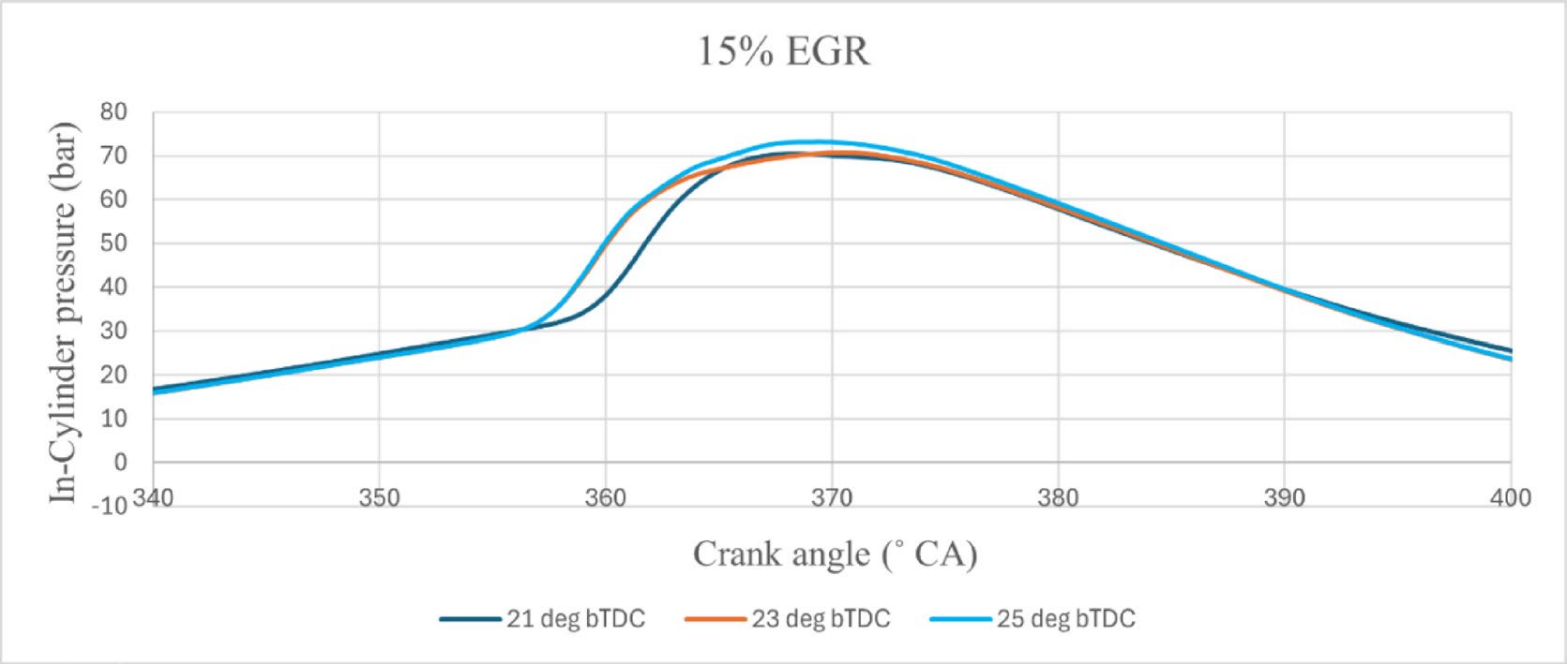
Peak CP vs CA at 80% load and constant speed = 1500 rpm for B25E15Zn50 blend at different IT of 21, 23, and 25°CA bTDC under (1) EGR = 0%, (2) EGR = 5%, (3) EGR = 10%, and (4) EGR = 15%.

The most effective technique for studying the combustion process is cylinder pressure monitoring. Figure 2 shows the variations of peak CP vs CA for B25E15Zn50 blend at various IT and under EGR rates of (1) 0%, (2) 5%, (3) 10%, and (4) 15%. Peak values of peak CP at various IT and EGR rates are shown in Table 7. The figures shows the 25° bTDC early injection of the B25E15Zn50 blend provide the combustion centre closer to TDC, increasing the peak CP. The delay time decreased when the IT were reduced from retard IT (23° bTDC to 21° bTDC). The IT from 23° bTDC to 25° bTDC causes combustion events closer to the TDC position, with greater peak CP and temperature, allowing for quicker fuel ignition^37,38^. Conversely, more delay in CD from 23° bTDC and 25° bTDC allowed the pre-combustion events to take place after the TDC position when the peak CP and temperature are high, therefore causing a longer ID. The increasing EGR rate from 5 to 15% extended the ID at any given IT. The inert gases in the exhaust emissions retarded down the pace of the chemical reaction and delayed the beginning of combustion. The elevated peak CP inside the cylinder, The introduction of EGR into the system results in a significant rise in maximum pressure, with recorded maximum pressures of 72.67 bar, 80.13 bar, 79.01 bar, and 78.5 bar for EGR rates of 0%, 5%, 10%, and 15% with various IT respectively.

**Table 7:** Pressure values of peak CP at 80% load.

IT (° bTDC)	EGR rate (%)	Peak CP (bar)
21	0	65.32
23	0	70.18
25	0	68.67
21	5	78.50
23	5	79.01
25	5	80.13
21	10	73.57
23	10	74.15
25	10	75.82
21	15	70.86
23	15	71.34
25	15	72.16

**Fig. 3:**
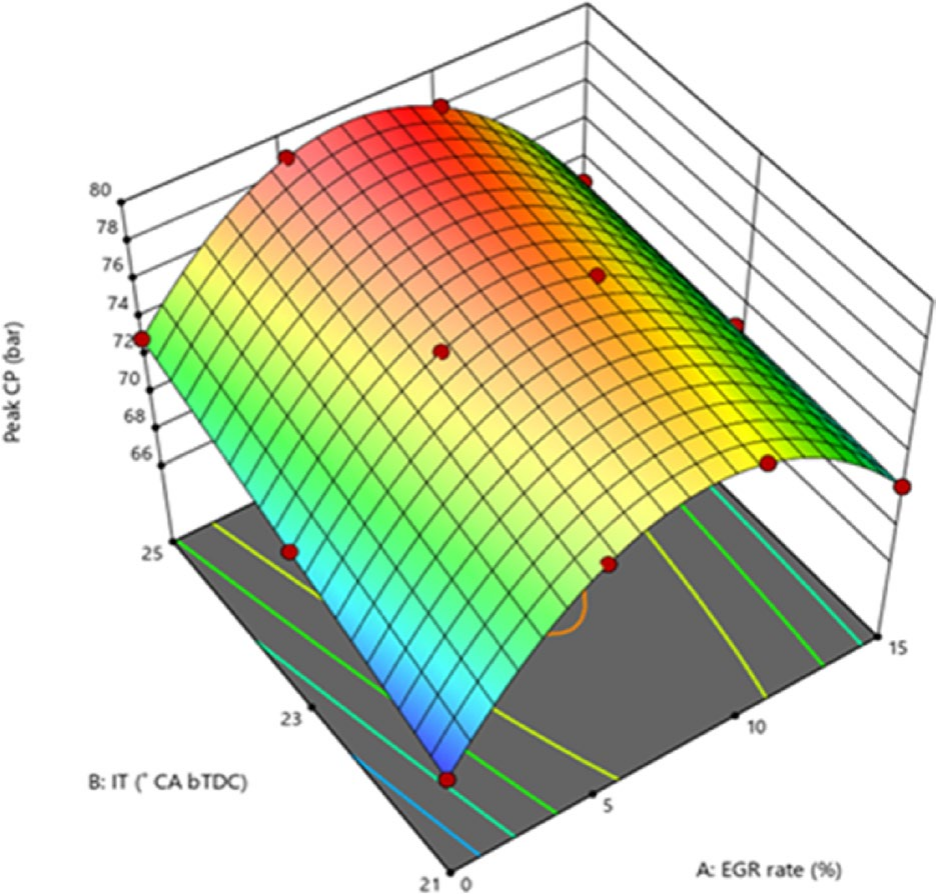
Predictive peak CP with different IT and EGR rates.

The addition of burnt exhaust gases to the intake air permits the mixture to have a high specific heat capacity. This mixture will absorb heat inside the combustion chamber, lowering the flame temperature. A greater rate of EGR reduces the combustion temperature, which lowers the pressure within the cylinder. Delaying the IT from retard IT causes a decrease in peak CP at a 5% EGR rate. In the case of an EGR rate of 10% and 15% in particular. The reason for this happens is that the fuel was able to undergo combustion with less amount due to the late injection, which led to a lower % of combustion at constant volume and greater peak CP^39^. The fundamental cause for the maximum peak CP at 25° bTDC is that more fuel accumulates at advanced IT, resulting in a quicker burning rate during the premixed combustion phase^40^. This occurs because, with retarded IT, the initiation of injection is postponed, resulting in a reduced CD, which subsequently decreases the peak CP. This aligns with the reduced NOx levels, which clearly indicate decreased in-cylinder temperatures that inhibit NOx formation. At an advanced IT of 25° bTDC, the temperature and pressure within the combustion chamber are slightly elevated, which influences the ID characteristics and results in minimal O_2_ availability for combustion, causing delayed combustion and consequently higher in-cylinder temperature^41^. Due to a longer ID, most combustion occurs in expansion stroke, which is not usable energy. Advanced IT reduce ID and push the peak nearer TDC, which is useable energy. At 5% EGR when injection is delayed, fuel injection occurs around TDC, when fuel preparation time is reduced, and most fuel goes unburnt in expansion stroke and out via exhaust gas. Early injection improves combustion over standard fuel injection. This is because standard IT takes longer to prepare fuel, which increases premixed combustion, when just a little amount of fuel is consumed and the combustion transitions to diffusion combustion. The buildup of unburned HC from premixed combustion into diffusion combustion causes incomplete combustion and reduced combustion. When the injection is advanced, there is adequate time for fuel preparation and appropriate combustion happens in the relevant phase likewise, the oxygenated nature of nano additions promotes the combustion^42,43^. The catalytic effect of NPs improved flame propagation speed while decreasing the temperature needed for fuel–air reactions. Furthermore, a larger S/V ratio of the NPs results in a homogenous air–fuel combination. The catalytic effect and homogenous distribution of the A/F mixture combine to provide effective combustion, resulting in a rise in exhaust gas temperature. Figure 3 illustrates the effect on predicted peak CP with function of various EGR rates and various IT.

#### Heat release rate

**Fig. 4:**
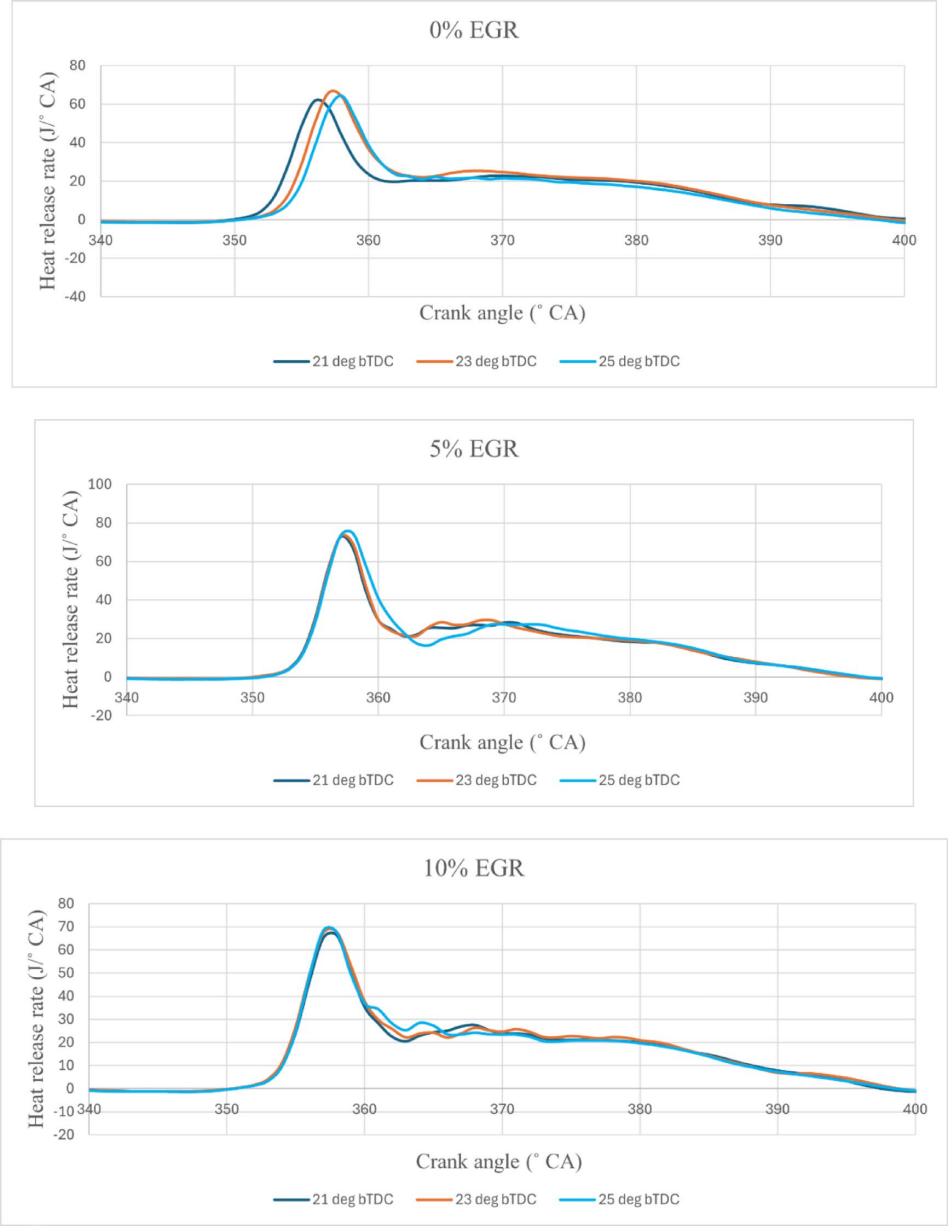

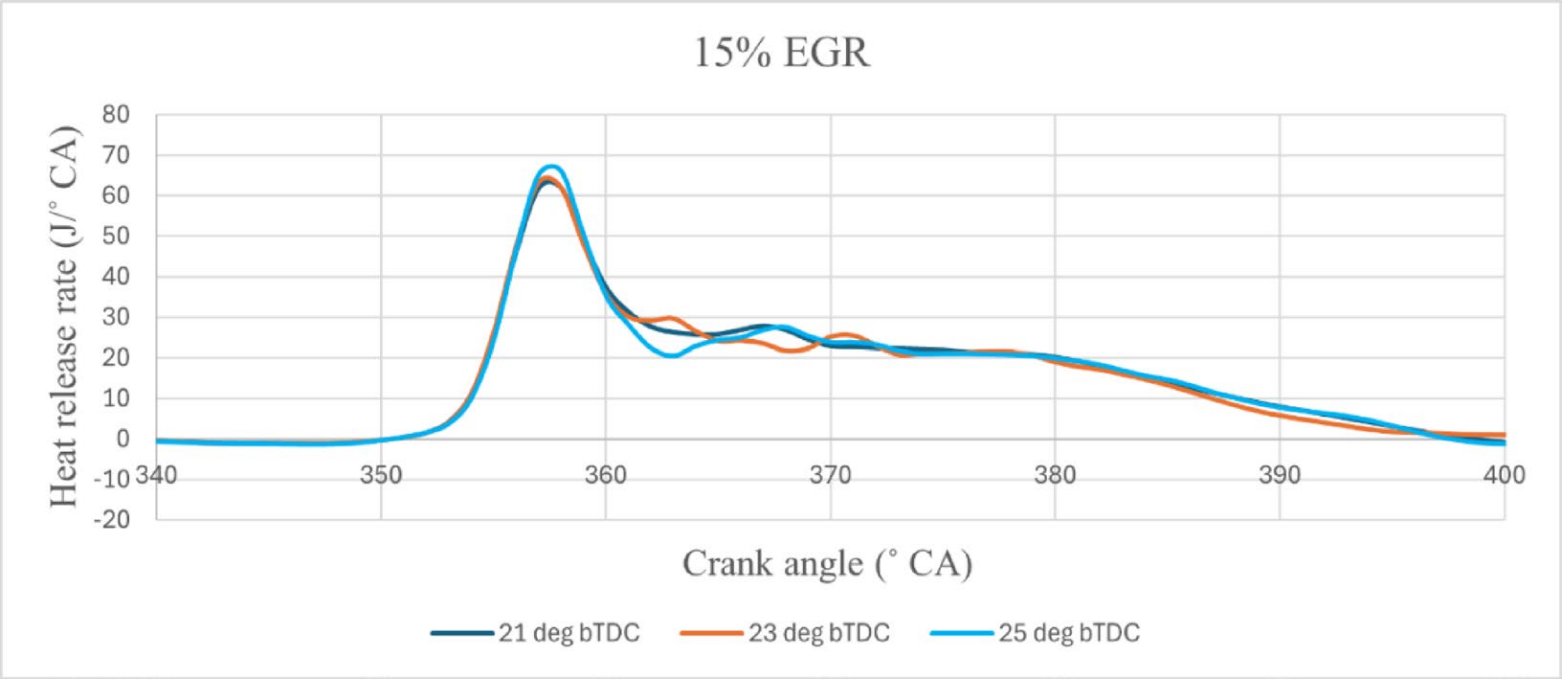
HRR vs CA at 80% load and constant speed = 1500 rpm for B25E15Zn50 blend at different IT of 21, 23, and 25°CA bTDC under (1) EGR = 0%, (2) EGR = 5%, (3) EGR = 10%, and (4) EGR = 15%.

The HRR refers to the pace at which energy is released from the fuel during combustion. A DI diesel engine’s combustion process is divided into two phases: premixed and diffusion. The first law of thermodynamics is used to calculate the rate of heat emission. Figure 4 represents the variations of HRR vs CA for B25E15Zn50 blend at different IT and under EGR rates of (1) 0%, (2) 5%, (3) 10%, and (4) 15%. The values of peak HRR at various IT and EGR rates are shown in Table 8.

**Table 8:** Peak Heat release values at 80% load.

IT (° bTDC)	EGR rate (%)	Peak HRR (J/° CA)
21	0	63.40
23	0	68.3
25	0	67.59
21	5	67.64
23	5	74.33
25	5	75.80
21	10	66.54
23	10	69.19
25	10	71.35
21	15	63.89
23	15	65.61
25	15	68.16

**Fig. 5:**
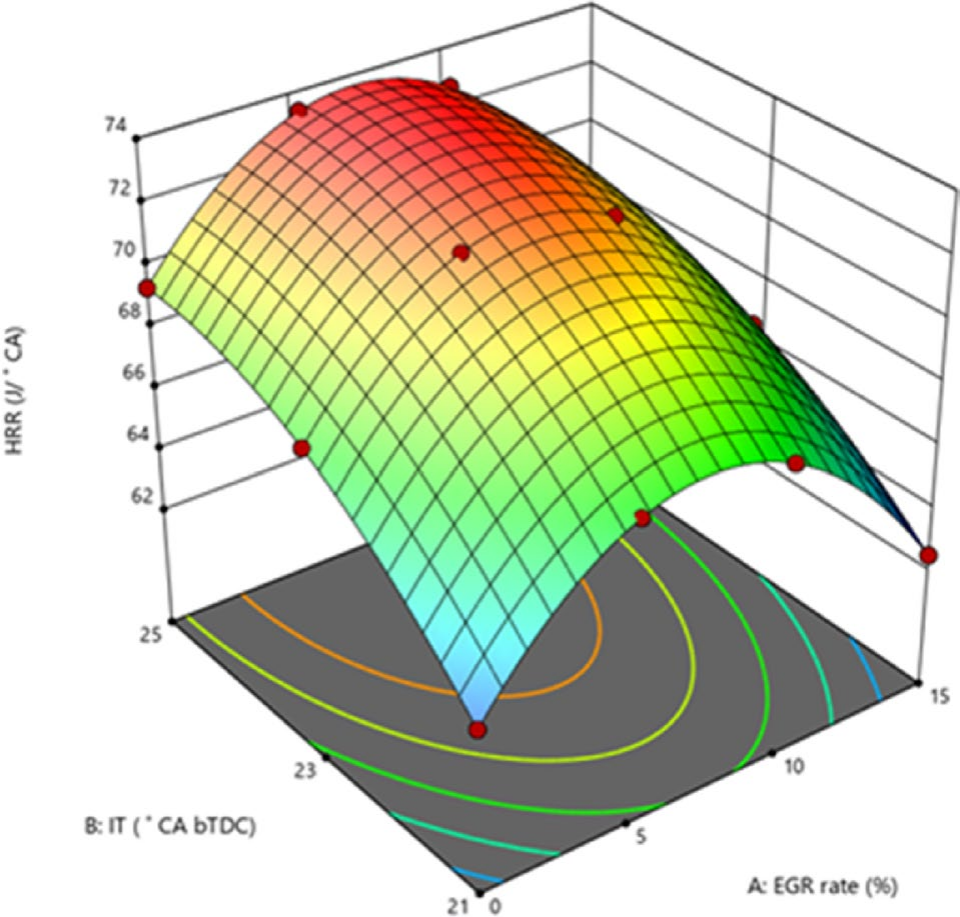
Predictive peak HRR with different IT and EGR rate.

The figures illustrate the 25° bTDC early injection of the B25E15Zn50 blend provide the combustion centre earlier CA prior to TDC, increasing the HRR. The main cause for 25° bTDC having the greatest HRR might be enhanced fuel spray characteristics, followed by better fuel–air mixing and a longer premixed combustion time, resulting in a higher residence rate. The enhanced CN characteristics of the B25E15Zn50 blend facilitate heat release at earlier CA, prior to TDC, resulting in an extended diffusion combustion phase, while the influence of O_2_ molecules is significant primarily in the latter phases of combustion^44,45^. The longer ID and better-oxygenated conditions in the B25E15Zn50 blend boosted flame speed during combustion, leading to a larger HRR. The HRR curves shift to the right when the IT is dropped, that change even more as the EGR rate rises. Between 23° bTDC and 21° bTDC, higher HRR diminished as IT increased. HRR was found to be higher when IT was postponed from 23° bTDC to 25° bTDC. Rising the IT from retard IT minimises the delay time and fuel usage during the premixed combustion phase, resulting in fewer HRR values. Further delay in the IT increased the delay period, enabling a greater quantity of fuel to burn during the premixed phase, resulting in a higher HRR. Increasing the EGR rate from 5 to 10% maximises the HRR. At a IT of 25° bTDC, HRR increased by 5%^46^. Figure 5 illustrates the effect on predicted peak HRR with function of EGR rates and various IT.

### Performance characteristics

#### Brake thermal efficiency

**Fig. 6:**
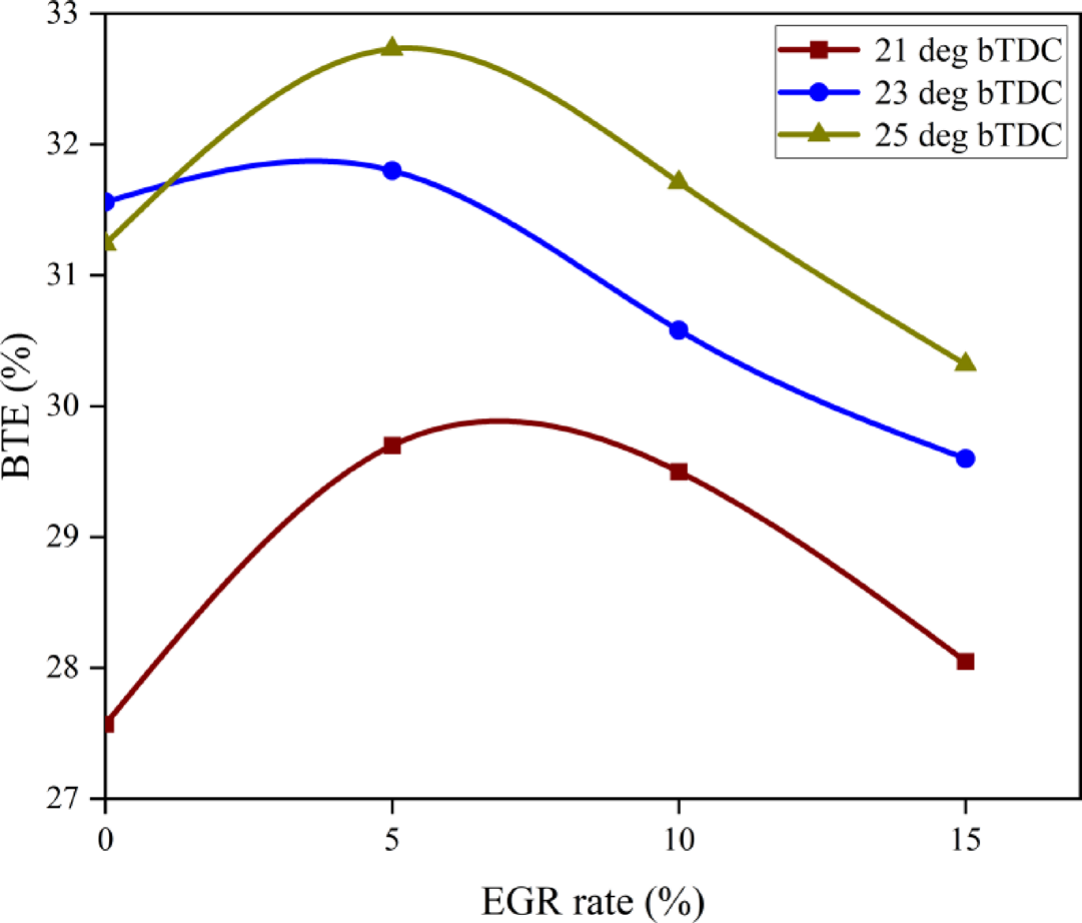
Effect on BTE with function of IT and EGR concentrations.

BTE measures how efficiently fuel is utilised during the combustion process to generate power output. The use of lower CV fuels during combustion has a direct influence on BTE. Figure 6 demonstrate the variance in BTE for the B25E15 blend with 50 mg/l ZnO nano addition at various IT and various EGR rates. The greatest BTE was recorded for 25° bTDC (32.73%), followed by 31.7% for 23° bTDC and 30.30% for 21° bTDC at 5% EGR at 80% load. Lowered BSEC with retarded IT were in line with the findings of Agarwal et al.^45^. The result can be ascribed to efficient fuel utilisation at 25° bTDC, which aligns well with the BTE profile, since 25° bTDC guarantees the greatest BTE throughout 80% load conditions. The efficient utilisation results from optimised IT, which enhances the performance of the B25E15Zn50 blend by increasing BTE and reducing BSEC profiles. Additional retardation in ignition timing to 21° bTDC led to increased BSEC levels. Previous research by Sengupta et al. revealed elevated BSEC with advanced IT^47^. The 25° bTDC IT increases the rate of complete oxidation and generates more heat with the same amount of fuel.

**Fig. 7:**
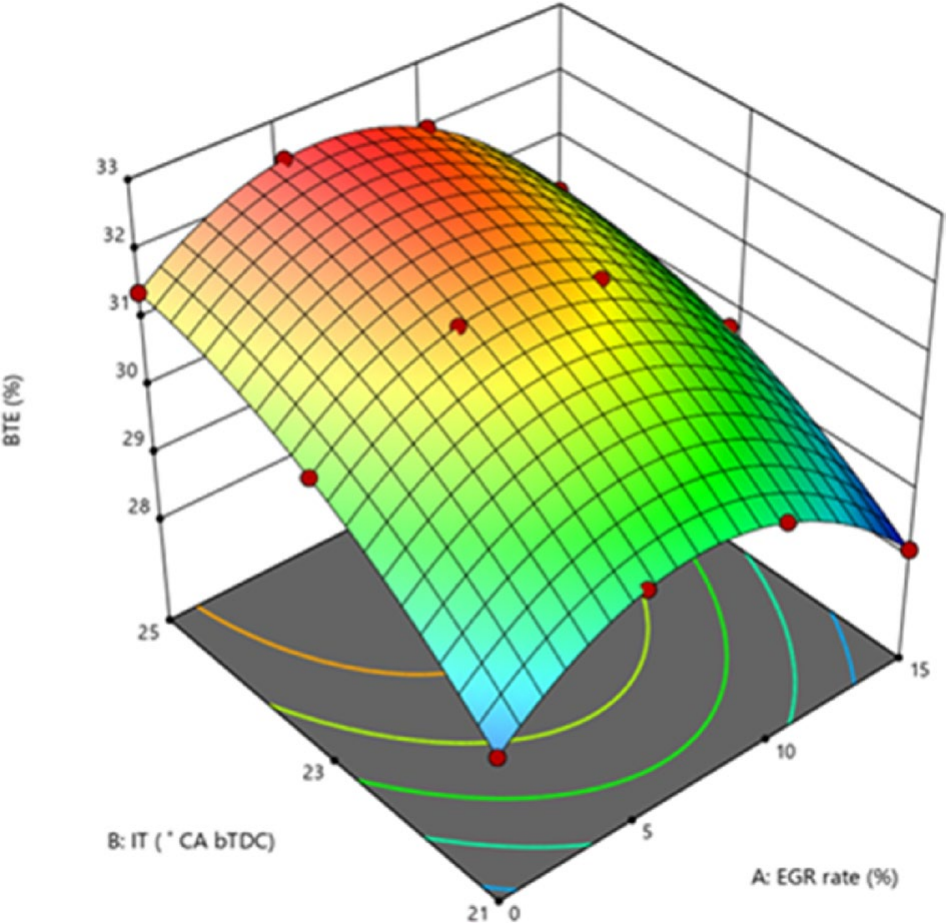
Predictive BTE with different IT and EGR rates.

Because combustion starts closer to TDC, at higher temperatures and pressures, this engine’s IT causes the ID to be shorter. An increase in both effective pressure and BTE results from igniting more quickly. When the engine is under greater load, the BTE increases up. Adding ZnO NP to the B25E15 blend boosts the BTE. The addition of NPs to the fuel enhances the S/V ratio, resulting in more reactive surfaces. This, in turn, accelerates the response rate of combustion, promotes complete combustion, and results in improved combustion efficiency. The low EGR rate and 25° bTDC showed a greater BTE. The HRR study provides support for this. Injecting the B25E15Zn50 blend at 25° bTDC causes a more concentrated rate of HRR, which ultimately produces more positive work and a greater BTE. At a high EGR level, lowering the BTE is achieved by increasing the EGR rate from 10 to 15%. The standard combustion process is hindered by a greater rate of exhaust gases, which affects the combustion efficiency^48,49^. Figure 7 indicate the effect on predicted BTE with function of EGR rates and various IT.

#### Brake specific energy consumption

**Fig. 8:**
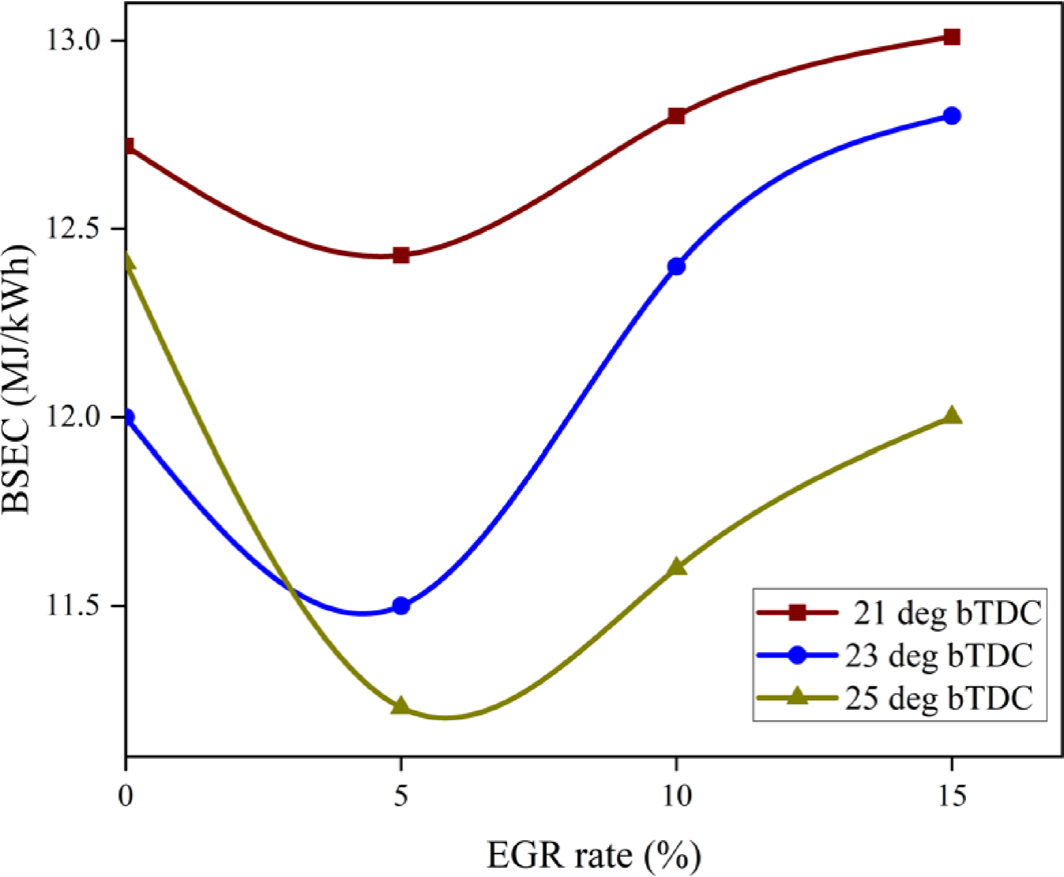
Effect on BSEC with function of IT and EGR concentrations.

**Fig. 9:**
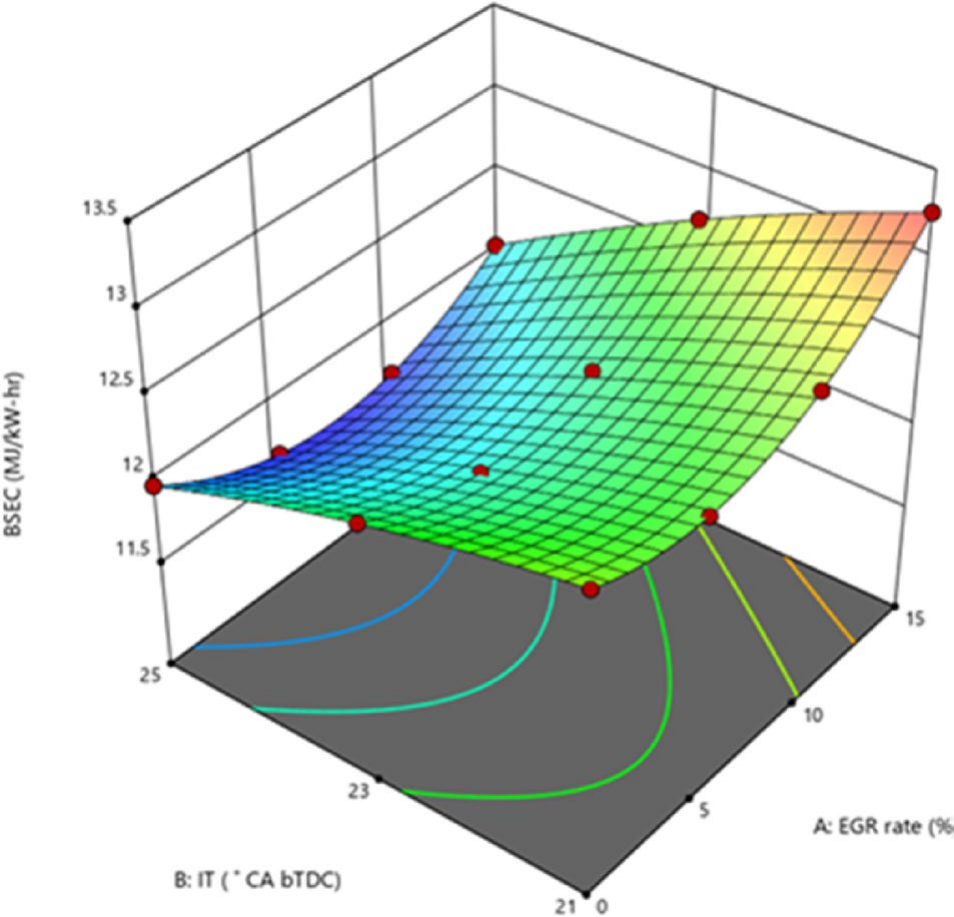
Predictive BSEC with different IT and EGR rates.

BSEC is a more accurate parameter for estimating engine performance characteristics than BSFC, as it considers the CV and density of the various fuels. Moving the IT ahead of timing from 23° bTDC to 25° bTDC decreases the BSEC. On average, IT injected at 23° bTDC and 25° bTDC were less than those injected at 21° bTDC as shown in Fig. 8. The BSEC increased in simultaneously with the proportion of EGR rates. It takes more fuel to keep the engine speed constant when exhaust gases and fresh air are injected into the combustion chamber, generating an O_2_-deprived environment on the inside. The B25E15Zn50 blend has a lower BSEC than diesel, which is likely because to its lower viscosity and higher CV^50^. Between 23° bTDC and 25° bTDC, during the delayed FIT, the combustion event occurred close to the TDC position, leading to complete combustion and a decrease in energy required to achieve the rated speed. By lowering the FIT from 21° bTDC, the combustion process moves to the expansion stroke, when heat loss occurs, resulting in less power output and more BSEC. A greater EGR level is achieved by driving the higher BSEC up from 10 to 15% EGR. This is because the EGR diluted the fuel–air mixture, which changed the burning rate and increased BSEC by decreasing the air–fuel ratio^51^. Figure 9 represents the variance in predicted BSEC with respect to IT and EGR rates.

### Emissions analysis

#### Carbon monoxide emissions

**Fig. 10:**
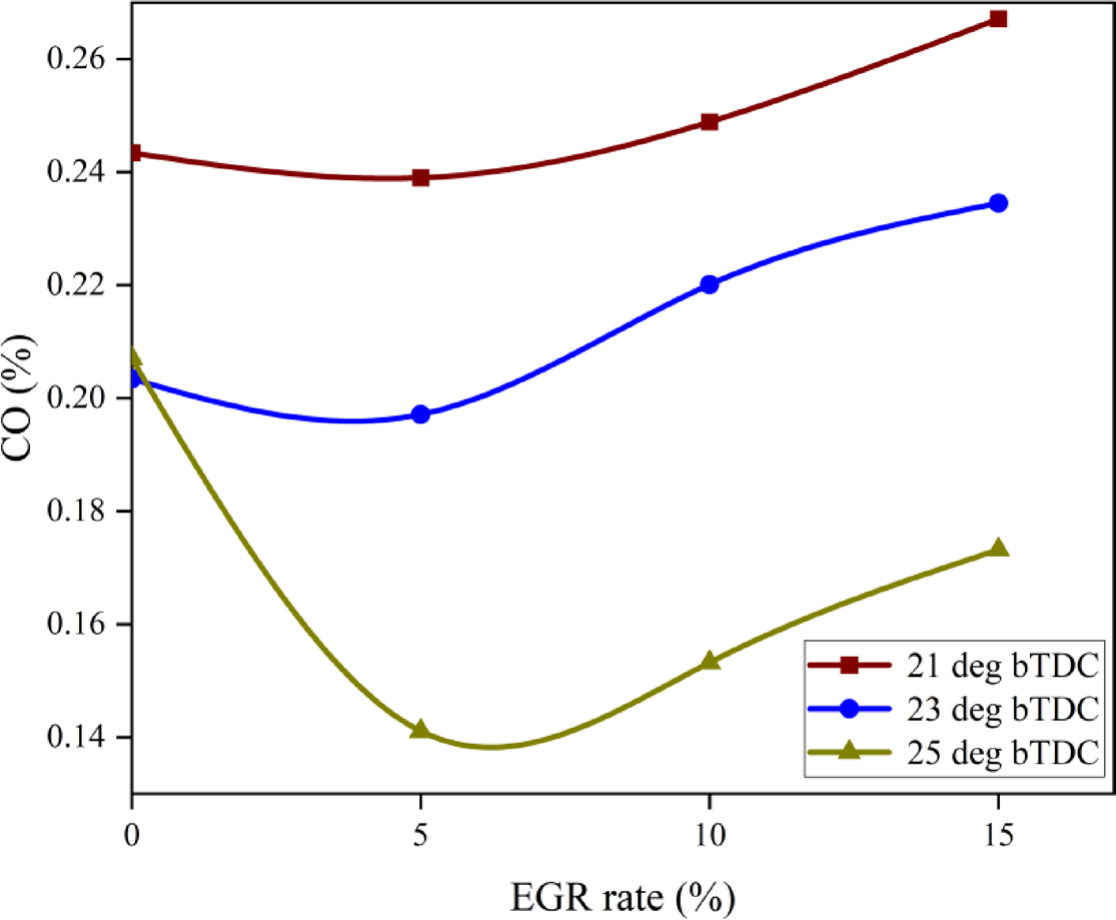
Effect on CO with function of IT and EGR concentrations.

Lack of air in the combustion chamber is one of the main reasons of dangerous CO. CO, the most dangerous GHG, must be drastically reduced. If there is insufficient O_2_ available to fully burn every carbon atom into CO_2_, some carbon is released as CO. CO will also be caused by incomplete combustion, concentrated rich regions, and inadequate mixing. At elevated loads, the B25E15Zn50 blend CO at 25° bTDC was inferior to that at retard IT as shown in Fig. 10. The primary reason for this is that at 25° bTDC, the accelerated combustion rate during the premixed phase diminishes the CD to a lesser extent than retarding IT, which inhibit the combustion of ZnO NP and consequently limits the catalytic activity of NP in oxidising CO molecules to CO_2_, leading to negligible CO reduction, particularly under higher loads. Because the B25E15 blend contains 50 mg/l ZnO NP, that serves as a combustion enhancer and causes a quicker rate of combustion, the blend has lower CO than 21° bTDC IT.

**Fig. 11:**
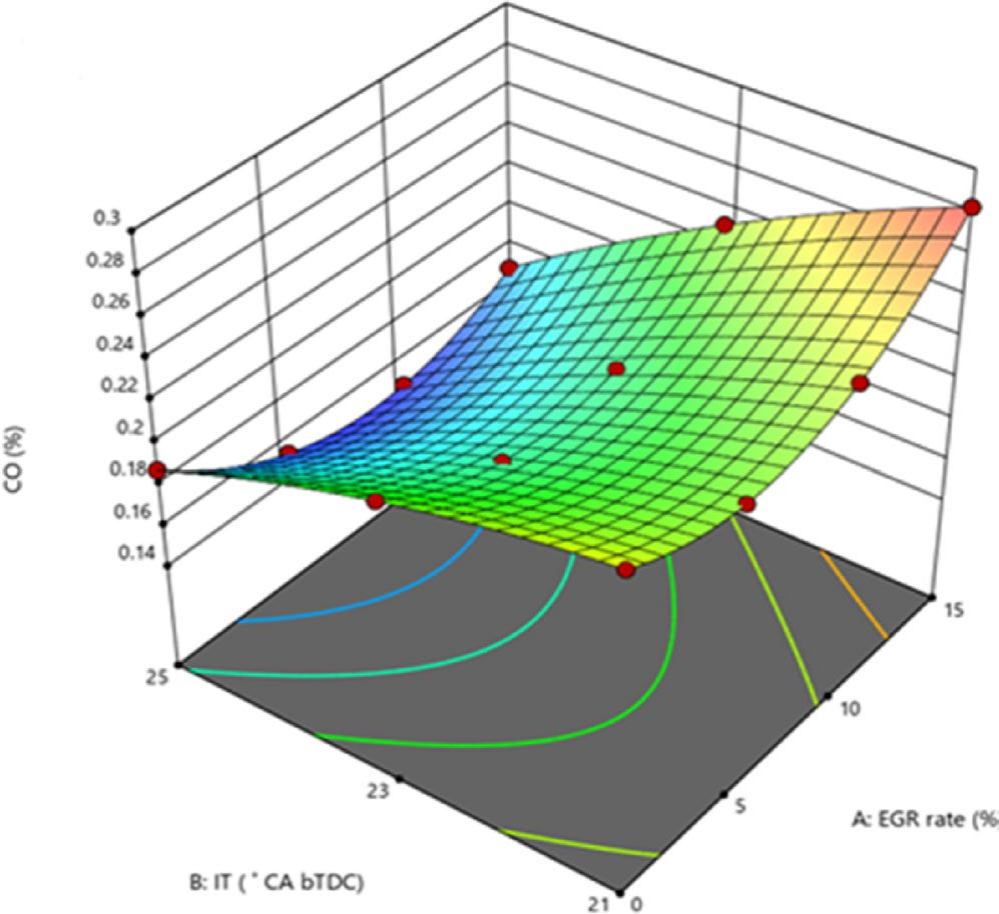
Predictive CO with different IT and EGR rates.

This is due to the fact that ZnO nano additives function as an O_2_ donor and buffer during the oxidation of CO molecules. Furthermore, ZnO NP’s high S/V ratio increases chemical reactivity and decreases ID, promoting complete combustion and lowering CO. Higher temperatures cause ZnO NP to dissociate, causing the ZnO molecule very unstable. Even if 21° bTDC does not increase engine temperatures or cylinder pressure, the inclusion of ethanol and NP with B25 blend promotes low temperature combustion(LTC). The inclusion of NP in the B25E15 blend works as a fuel-mixer binder and vaporises at LTC, resulting in a higher partial oxidation rate and more CO at practically all loads than advanced IT^52^. A delayed IT marginally raised CO, while greater EGR rates significantly reduced emissions. Such as At 5% EGR rate, IT was lowered from 25° bTDC to 23° bTDC. The CO increased due to a shorter ID, which gave the A/F mixture less time. However, boosting EGR reduced CO significantly. A lower air intake owing to the EGR addition created an O_2_-deficient environment in the combustion chamber, limiting CO oxidation. LTC lowered OH radical concentration, reducing CO-to-CO_2_ conversion. Since restarted fuel IT and greater EGR rates cause combustion at low temperatures, NOx and CO have an inherent trade-off relationship. Low temperature promotes incomplete fuel-to-CO_2_ conversion, limiting efficiency and increasing CO^53^. Figure 11 shows the effect on predicted CO with function of EGR rates and various IT.

#### Hydrocarbon emissions

**Fig. 12:**
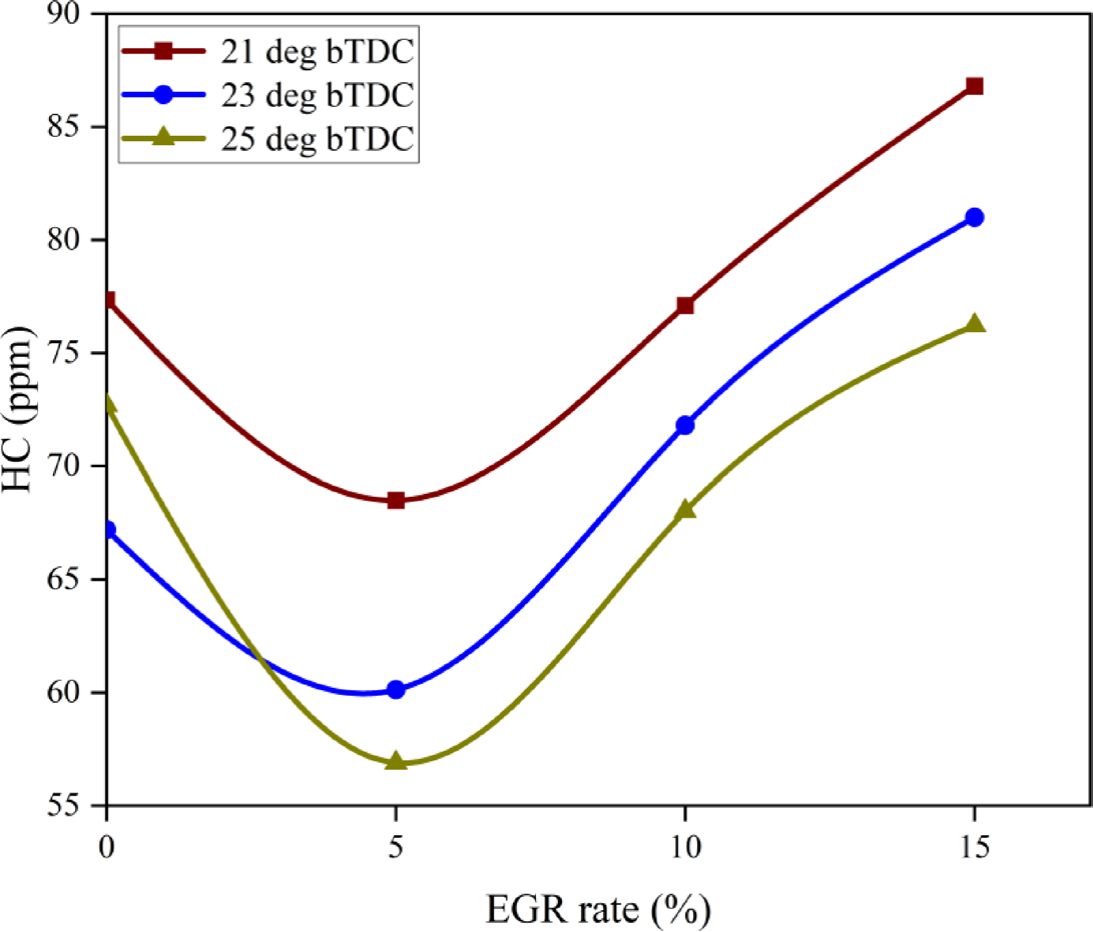
Effect on HC with function of IT and EGR concentrations.

**Fig. 13:**
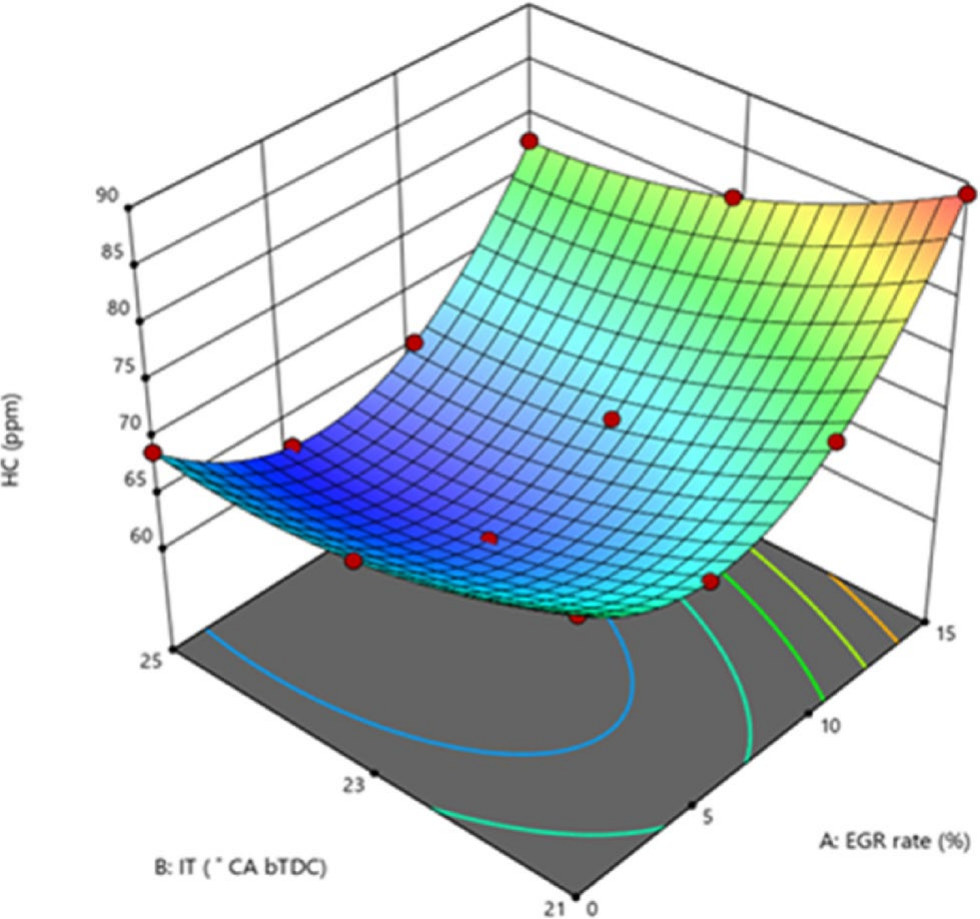
Predictive HC with different IT and EGR rates.

HC are the result of incomplete combustion of fuel products. Incomplete combustion happens for a variety of causes, including low fuel/air mixing efficiency, engine operating conditions, IT, and ID. HC is very infectious to human health, hence it is crucial to control the produced quantity during combustion. Common substitutes for reducing HC generation include NP and catalytic converters. Particularly in quench layers, the activation energy of ZnO NP often burns off carbon deposits inside the engine cylinder. Moreover, at higher temperatures, the in-built O_2_ atoms produced by nano additions assist to oxidise soot precursors, thereby promoting more complete combustion and lower HC. At maximum load, the B25E15Zn50 blend HC at 25° bTDC was lower than retard IT as shown in Fig. 12^54^. The fuel’s high rate of heat transfer is facilitated by the presence of an O_2_ buffer in nano additives, as well as by the fuel–air combination’s reduced viscosity and larger contact area. As an O_2_ giving catalyst, metal oxide NP provide O_2_ for the oxidation of unburned HC and CO. This contradicts earlier research showing that HC dropped with more sophisticated IT. The primary cause of this increase is because a longer ID results in a lower power output due to a slower rate of flame propagation. The HC in advanced IT at B25E15Zn50 blend is lower than in retard IT, owing mostly to the effects of secondary atomisation and oxidation^55^. EGR intensification from 10 to 15% increased HC considerably. The addition of exhaust gases lowered gas temperature, preventing HCs from splitting into elemental carbon particles and increasing HC^50^. Figure 13 indicate the effect on predicted HC with function of EGR rates and various IT.

#### Smoke emissions

**Fig. 14:**
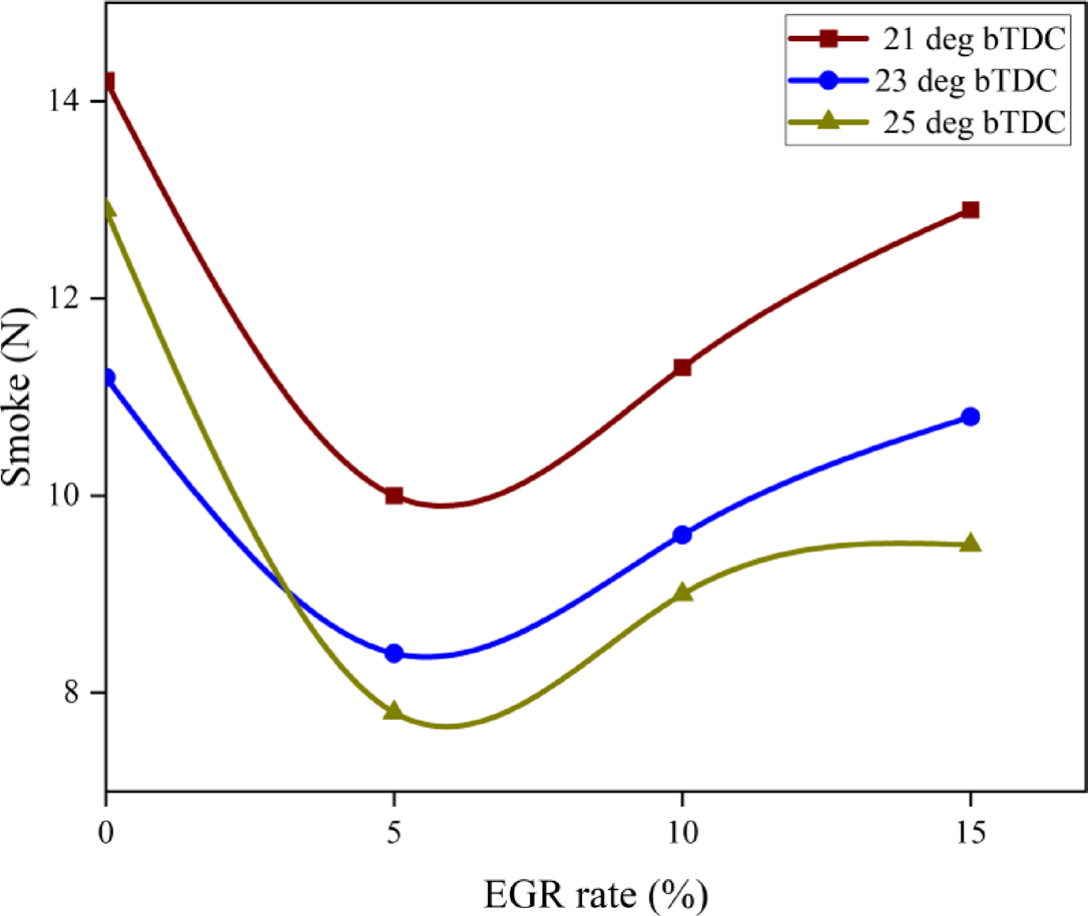
Effect on smoke with function of IT and EGR concentrations.

In most cases, incomplete combustion produces smoke. Poor atomization, too much fuel accumulating in the combustion chamber, and a lack of air in the combustion-rich zones are the main causes of smoke generation in CI engines. The B25E15Zn50 blend smoke at 25° bTDC were less than those at retard IT while it was operating at 100% load as shown in Fig. 14. When ZnO NP was added to the B25E15 blend, the evaporation rate increased, the ID decreased, and the ignition properties improved. Because of the shorter ID time, more fuel is gathered inside the combustion chamber before it ignites, improving air–fuel mixing and increasing combustion efficiency, which in turn reduces smoke. Due to the existence of optimal fuel viscosity and CN characteristics, which may have an impact on the droplet diameter size and fuel spray characteristics, 25° bTDC IT demonstrated a considerable reduction in smoke levels when compared to other IT. Furthermore, ID is reduced since there is less fuel accumulation, resulting in a slow burning rate, and a gradual rise in cylinder pressure and temperature does not improve ZnO NP catalytic activity^56^. The B25E15Zn50 blend, injected at 25° bTDC with 5% EGR, exhibits reduced soot compared to diesel injected at the same timing without EGR, due to its higher O_2_ concentration that facilitates soot oxidation during combustion.

**Fig. 15:**
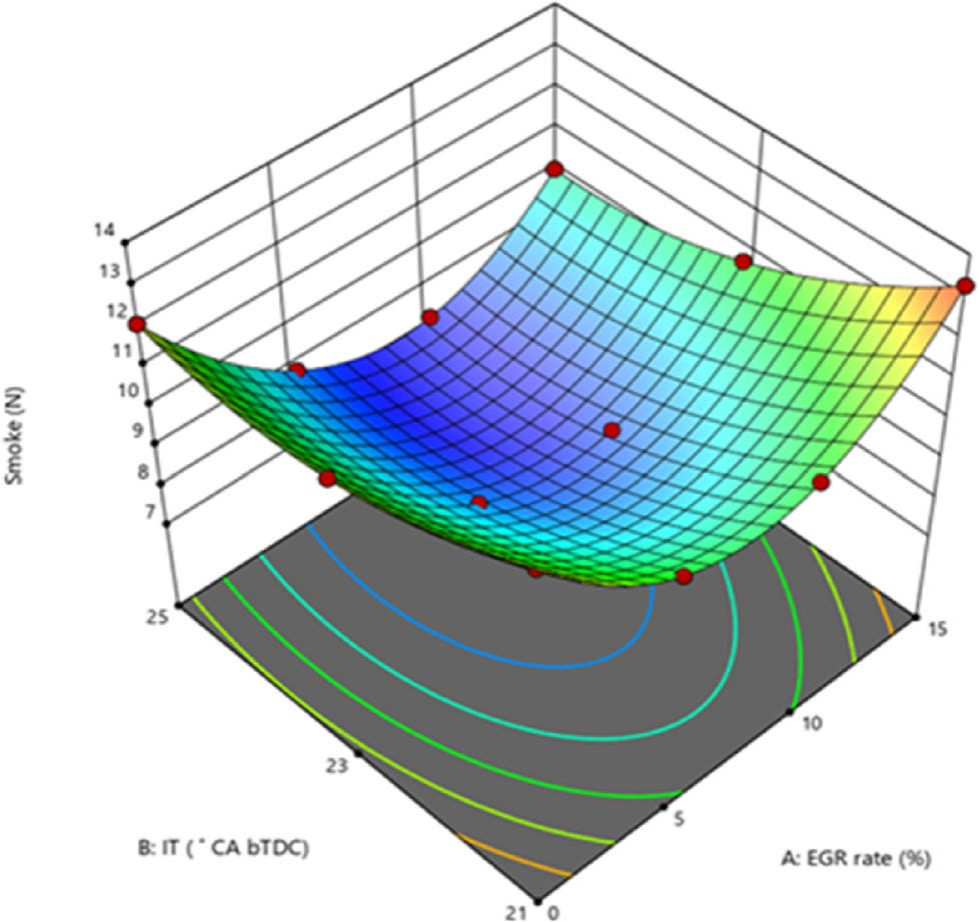
Predictive smoke with different IT and EGR rates.

The increased ID of the IT facilitates improved air–fuel mixing, resulting in a more homogeneous mixture. This leads to an enhanced premixed combustion phase and a subsequent diminished diffusion phase, hence decreasing smoke output. The smoke opacity increases as the EGR rates rise. The heat generated during combustion is trapped by gaseous species in the exhaust, lowering the cylinder temperature and inhibiting the oxidation of soot^57^. Figure 15 demonstrate the effect on predicted smoke with function of EGR rates and various IT.

#### Nitrous oxide emissions

**Fig. 16:**
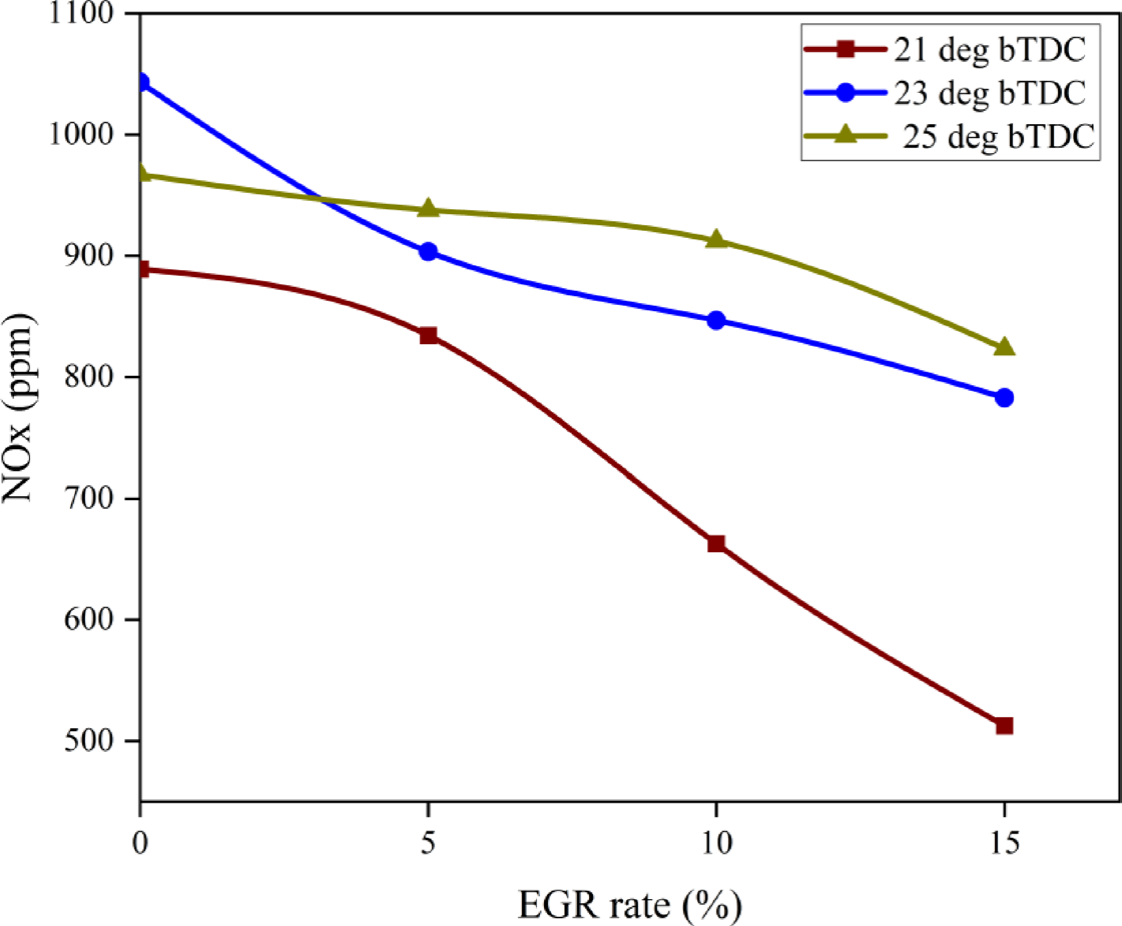
Effect on NOx with function of IT and EGR concentrations.

NOx and nitric oxide (NO) are the oxides found in the exhaust. At comparatively greater temperatures, nitrogen and O_2_ react. High temperatures and O_2_ availability are therefore the two primary causes of NOx production. More NOx is produced when there is more O_2_ available and the peak combustion temperature rises. Reduced NOx at 21° bTDC can be attributed to optimal engine operating parameters, such as optimal IT, CD, injection fuel spray, and reaction time^58^. The addition of NP in the B25E15 blend increases NOx in both 23° bTDC and 25° bTDC IT as shown in Fig. 16. However, very minor differences were noticed in the instance of 21° bTDC. At advanced IT, adding ZnO NP to B25E15 blend resulted in higher NOx than 21° bTDC IT at entire engine loads, respectively. Reducing NOx resulted from the IT between 25°CA bTDC and 21°CA bTDC. The majority of combustion events were postponed by late injection, resulting in a TDC, which ultimately lowers the combustion rate and stops the production of NOx. EGR is an effective method often used to reduce NOx for diesel engines. Increased EGR significantly lowers NOx. By reducing the O_2_ concentration and combustion temperature and efficiently increasing the exhaust gas’s heat capacity, EGR will prevent the production of NOx. Reduced NOx depends on a cooling impact imposed during the combustion process by higher latent heat of vaporisation feature of ethanol with ZnO NP blend. NOx was more than two-fold reduced when the EGR rate raised from 10 to 15% at a certain IT.

**Fig. 17:**
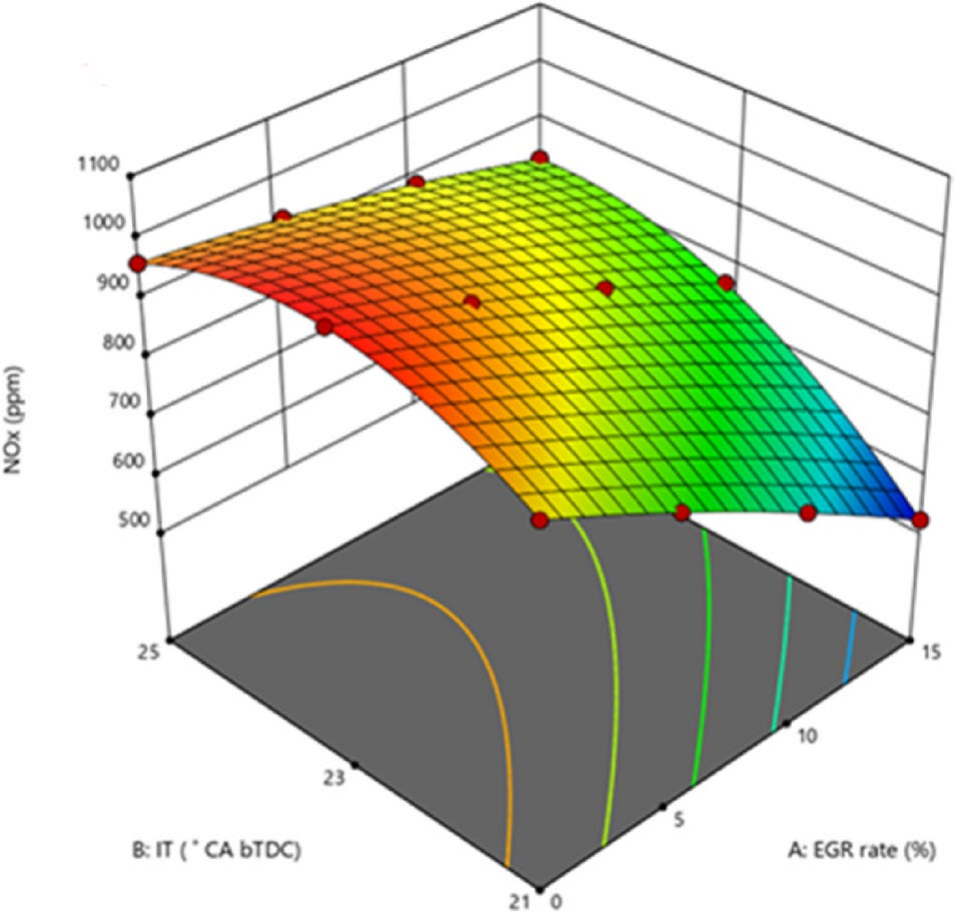
Predictive NOx with different IT and EGR rates.

The diluting effect of higher-specific-heat exhaust gases lowers gas temperatures. Chemical reaction rate was also impacted by O_2_ shortages^59,60^. This is demonstrated by the ideal IT, which lowers the NOx by lowering the maximum temperature of burning gases and the amount of O_2_. A little increase in NOx was observed when the IT was advanced to 25° bTDC as compared to 21° bTDC. This slight increase is caused by delay IT burning earlier than TDC, which raises cylinder pressure and HRR, accelerates the achievement of advanced IT, and raises the NOx profile. Figure 17 shows the effect on predicted NOx with function of EGR rates and various IT.

### Comparison of ANN and RSM model

**Fig. 18:**
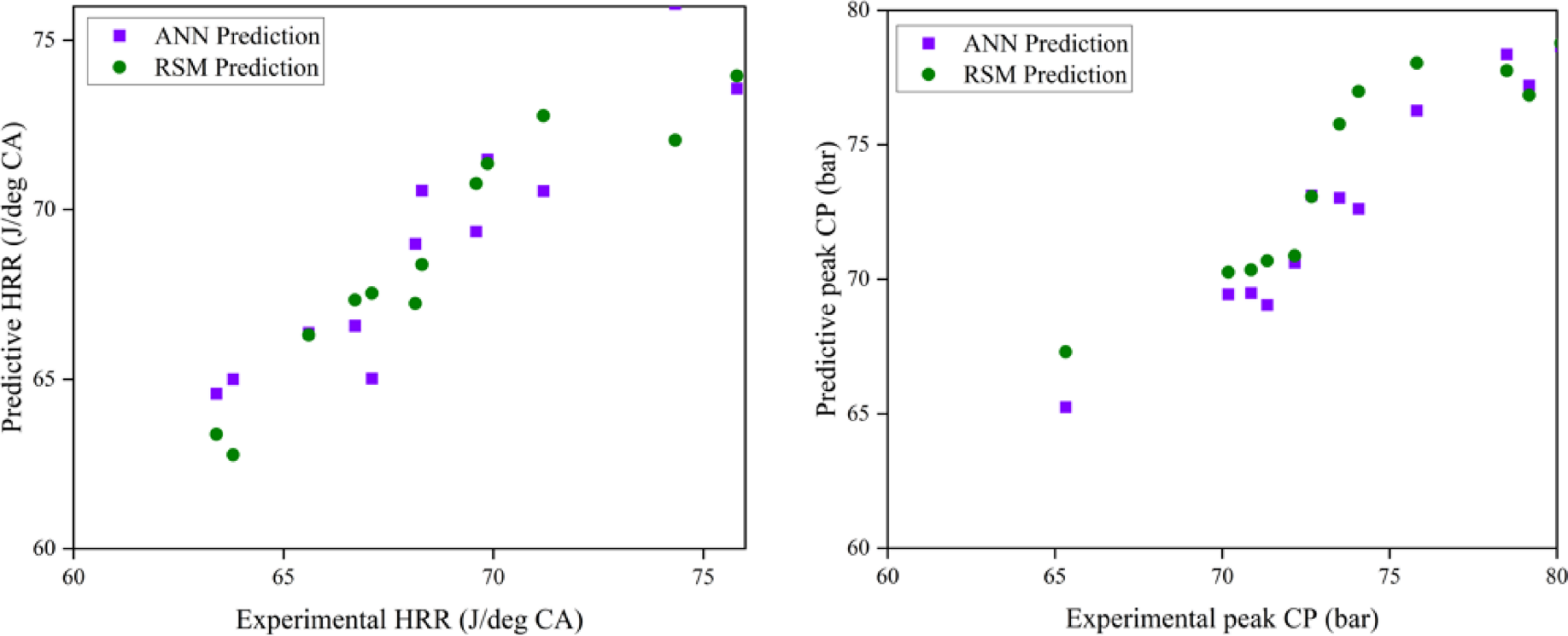
Variation of combustion parameters with experimental and optimization techniques.

**Fig. 19:**
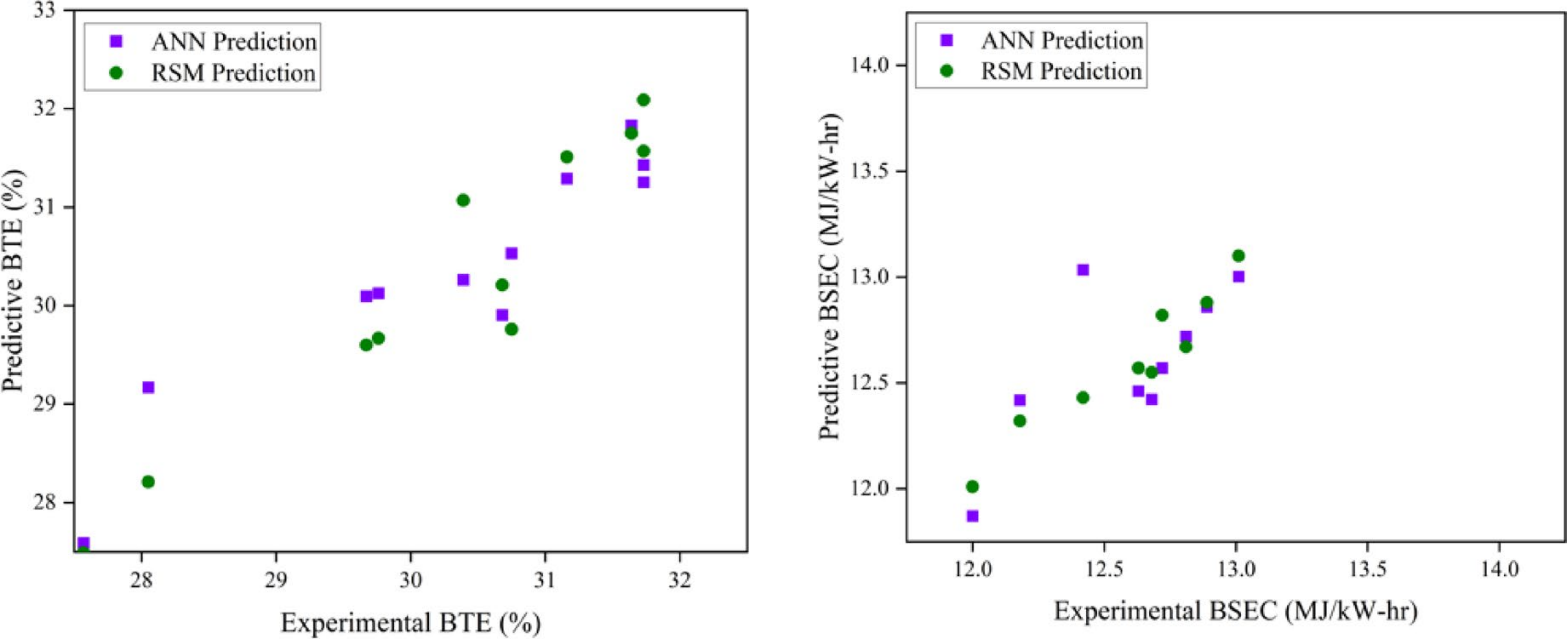
Variation of performance parameters with experimental and optimization techniques.

**Fig. 20:**
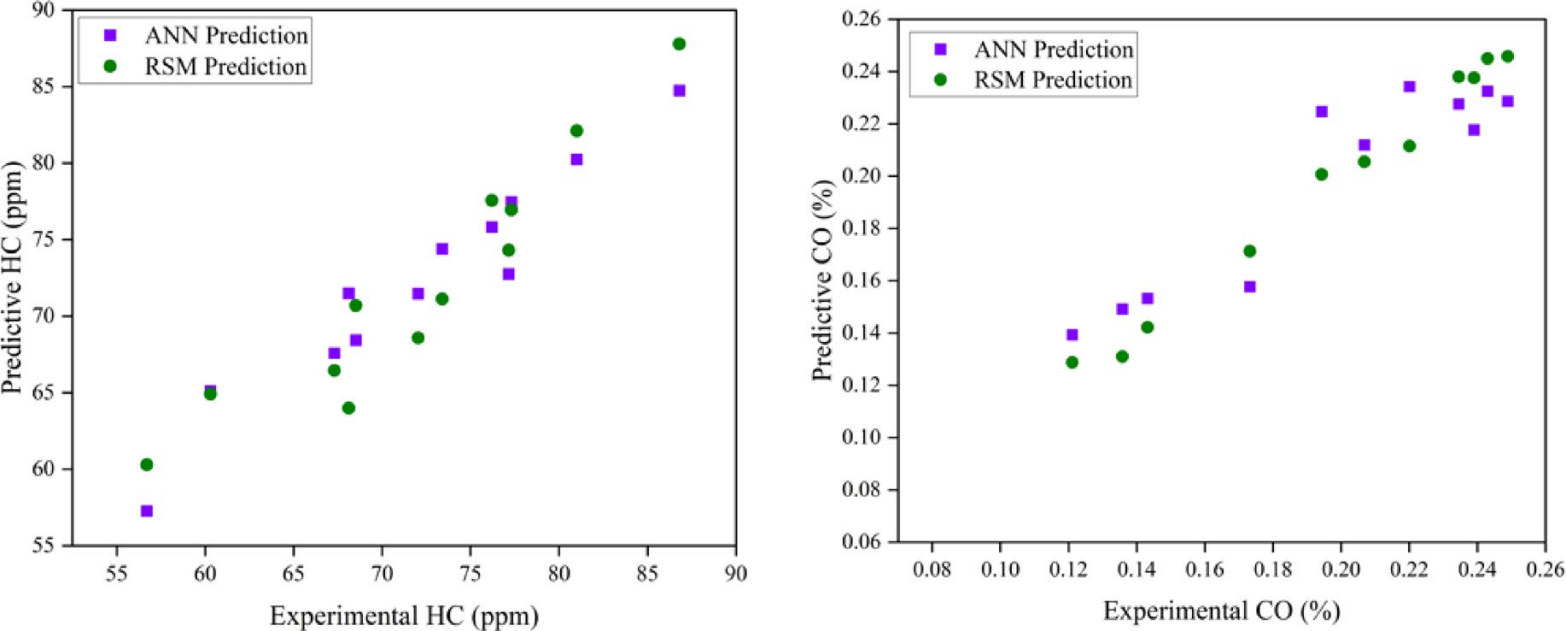
Variation of emissions parameters with experimental and optimization techniques.

**Fig. 21:**
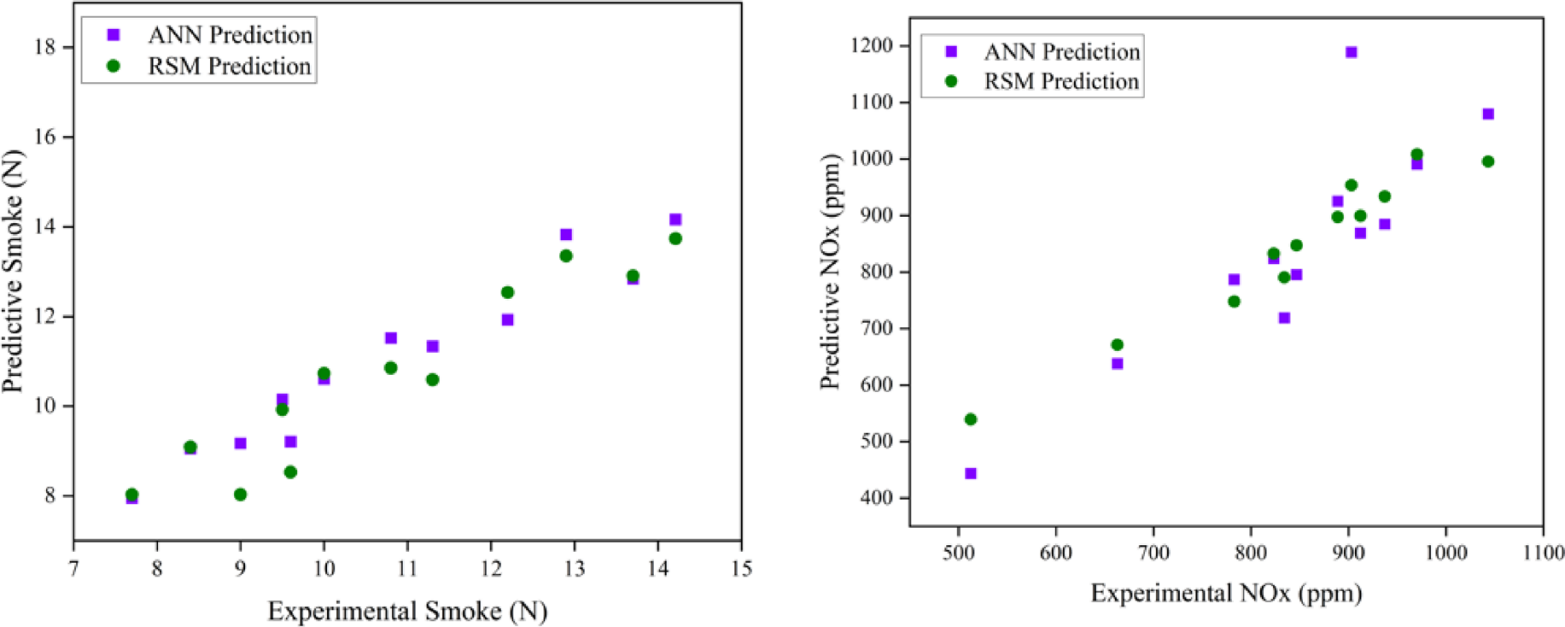
Variation of emissions parameters with experimental and optimization techniques.

Both models are able to anticipate the engine output responses, as evidenced by the predicted values being closer to the linear fit line. On the other hand, the RSM model’s predicted R^2^ were derived using the entire data’s actual and predicted values. The impact of input variables on the quality of their output response is evaluated using sensitivity analysis. This analysis is used to identify the ideal collection of experimental variables, hence reducing experimental training time, while the ANN model validates the RSM-predicted data using the R^2^ coefficient. The RSM and ANN models have R^2^ values of 0.979432 and 0.910079, respectively, suggesting their success as predictive tools for engine parameter responses^28^. This model is only applicable to this research, but it clearly opens the door to validating experimental optimisation findings using RSM or other optimisation methods. According to the author, the purpose of this research is to promote ANN as a standard for engine optimisation. The present event suggests that the ANN should be used to predict future engine operating behaviour. This also contributes to efforts to reduce the cost and time required for more experimental tests by identifying non-experimental values using ANN prediction and RSM optimisation. When comparing the R^2^ values of the two models, the RSM prediction performs better than the ANN prediction in terms of its correlation with experimental data. RSM performed better than ANN in terms of R^2^ and other significant error analysis metrics due to the prediction techniques. Consequently, the RSM model that was constructed was used for optimisation. In comparison to RSM-generated values, the ANN-derived projected values showed a stronger correlation with the experimental values (R^2^ > 0.95), suggesting higher prediction accuracy. In analysing the R^2^ values for both models, the RSM model has a higher R^2^ value, indicating a stronger correlation between the variables and superior predictive ability than the ANN model. This comparison illustrates the ANN model’s improved prediction ability and effectiveness over the RSM model^61^. The models were used to forecast a maximum point instead of fitting a curve to the experimental data, since the model generates predictive data. Utilising the developed ANN models, the untested interval points were analysed to determine the theoretical maximum location. The ANN modelling strategy is an effective method for addressing nonlinear issues. Consequently, it can be used to examine the relationship between inputs and outputs of experimental data. Consequently, an ANN was developed to assess engine performance and associated pollutants in this research. The goal of the optimisation is to suggest different IT and EGR rates for improved performance and reduced emissions with due regard to the weightage and importance given to each of the output responses. Choices with a higher desire score and a closer match to the provided criteria are favoured. Table 9 illustrates the weightage and importance based parameters. The optimized input parameters of IT of 26.4° bTDC and 8.63% EGR rate at B25E15Zn50 blend represent the most optimum output engine characteristics as shown in Table 10. Figures 18, 19, 20 and 21 represent the actual output and optimal predicted output.

**Table 9:** Constraints.

Name	Goal	Lower limit	Upper limit	Lower weight	Upper weight	Importance
A:EGR rate (%)	In range	0	15	1	1	3
B:IT (° CA)	In range	21	25	1	1	3
Peak CP (bar)	Maximize	67.306	78.777	1	1	3
HRR (J/° CA)	Maximize	62.766	73.949	1	1	3
BTE (%)	Maximize	28.210	32.598	1	1	3
BSEC (MJ/kWh)	Minimize	11.419	13.104	1	1	1
CO (%)	Minimize	0.129	0.269	1	1	1
HC (ppm)	Minimize	60	88	1	1	1
Smoke (N)	Minimize	8.034	13.741	1	1	5
NOx (ppm)	Minimize	539	1008	1	1	5

**Table 10:** Experimental validation.

Parameters	Experimental engine operating data	RSM prediction data	Error (%)	Mean absolute Percentage Error (MAPE)
Peak CP (bar)	80.13	81.47	1.67	22.1875
HRR (J/° CA)	75.8	77.10	1.70	
BTE (%)	32.7	33.12	1.32	
BSEC (MJ/kWh)	11.31	11.02	2.5	
CO (%)	0.1609	0.1583	2.6	
HC (ppm)	56	55	3.2	
Smoke (N)	7.8	7.64	2.05	
Nox (ppm)	780	757	2.97

### Prediction capability

A large number of tests were carried out with different input parameter levels, EGR rates (5, 10, and 15%), and fuel IT (21° bTDC, 23° bTDC, and 25° bTDC). In order to minimise the impact of random error, the experiments were conducted three times for each measurement. A database was created from the gathered replies. An RSM and ANN model was developed to anticipate output responses using all of the output data. The TRAINLM function, commonly known as the Levenberg–Marquardt back propagation technique, was applied to the framed network. The TRANSIG method helps to determine the output parameter depending on the input variable. The appropriate neurone size of ten was established via trial and error. The ANN model also conducted recurrent training, testing, and validation on each of the replies. The best match is achieved with engine emission and performance when the ANN model estimations are taken into account. Engine characteristics are the most closely matched responses with experimental data, according to RSM model estimates. It would seem that RSM has excellent modelling abilities and performance. In contrast to the ANN model’s R^2^ values ranging from 0.88 to 0.98, the constructed RSM model exhibits R^2^ values over 99%. Compared to the ANN model, the RSM model achieves superior results. Based on the results of the optimisation and modelling processes, it is evident that RSM outperformed ANN with respect to R^2^ and other metrics used for error analysis. The RSM was proven to be highly useful in observing the influence of several variables on engine performance and exhaust emissions. Both ANN and RSM models were trained using DoE contrasts. Comparisons used average % error and other characteristics. RSM predicts more deviation than ANN. This investigation concludes that the engine’s overall performance and prediction are enhanced by the superior precision accuracy provided by both RSM and ANN models. ANN and RSM are viable tools for predicting engine performance and have the potential to enhance the reliability of optimisation through improved correlations.

### RSM and ANN modelling approach for prediction

The objective of the current research is to examine the effects of varying EGR rates with variable IT in a ternary mix fed by a ZnO NP-fuelled CI engine. This investigation would clarify the combustion of engine parameters in CI engines and the resultant emissions produced. This study effectively used predictive models (RSM and ANN) to enhance outcomes and anticipate input parameters. The use of these technologies significantly decreases the time and costs associated with experimenting while enhancing the system’s efficiency. To develop the optimal empirical model, the operational function of RSM serves as a statistically based investigation that effectively establishes the interactions among various components. This research used RSM to develop a regression model and determine the optimal engine operating parameters with ideal ternary blend and ZnO NP proportion. Furthermore, an ANN model was constructed to enhance the precision of predictions about the reaction under varying load situations with different engine operating techniques. The best experimental variables are chosen using this approach, which shortens the experimental training period. The ANN model then verifies the RSM-predicted data using the correlation coefficient. A comparative analysis of the engine parameters of the ANN and RSM models. The anticipated values align more closely with the linear fit line, suggesting that both models effectively forecast engine output responses. The R^2^ value in the ANN graph relate only to the testing situation. For the RSM model, R^2^ values were derived from the actual and expected results of the comprehensive dataset. The mean R^2^ value for ANN engine performance was 0.9830, and the RSM value was 0.9994. Consequently, the predictive methodologies indicated that RSM exhibited superior performance compared to ANN in terms of R^2^ and other pertinent error analysis metrics. A validation test is required, in addition to optimisation, to assess the correctness of the obtained findings. A test was performed under the optimised conditions for validation.

## Conclusion

To determine the optimal operating parameters for the diesel engine, two alternative modelling software modelling were compared and tested. The optimized B25E15Zn50 blend is forecasted using the RSM and ANN models, with input factors including IT of 21°, 23°, and 25° bTDC and various EGR rates. The experimental conditions employed DOE methods with a CCD to estimate the minimal number of runs and levels required to develop an RSM model.

Advancing IT resulted in improved performance and emissions but at the cost of increased NOx. Increased EGR rate reduced performance and emissions. An IT and EGR rate that results in optimal engine output responses is evaluated by assigning due weightages to each of the responses.

The established engine operating conditions of 26.4° bTDC, 8.63% EGR rate resulted in improvement of peak cylinder pressure (CP), heat release rate (HRR), brake thermal efficiency (BTE) by 12.3%, 9.9%, 3.7% respectively and also reduction in hydrocarbon (HC), carbon monoxide (CO), smoke, and nitrogen oxides (NOx) by 26.4%, 19.6%, 43.6% and 33.7% respectively at 80% load.

This research is a comparative analysis of two methodologies. For this purpose, experimental test metrics were used for the modelling of RSM and ANN. Subsequently, these two models were evaluated against the test data. The RSM proved to be very useful for analysing the impact of various variables on engine characteristics. According to the results of the experiments, an advanced IT with low rate of EGR showed remarkable promise as a feasible alternative. This approach could contribute to improve the country’s energy security by lowering dependence on expensive and reducing fossil fuel reserves.

## Data Availability

The datasets used and/or analysed during the current study available from the corresponding author on reasonable request.

## References

[1] Wang, Q., Wang, L. & Li, R. Renewable energy and economic growth revisited: The dual roles of resource dependence and anticorruption regulation. *J. Clean. Prod.* **337**, 130514. 10.1016/j.jclepro.2022.130514 (2022).

[2] Umar, M., Ji, X., Kirikkaleli, D. & Alola, A. A. The imperativeness of environmental quality in the United States transportation sector amidst biomass-fossil energy consumption and growth. *J. Clean. Prod.* **285**, 124863. 10.1016/j.jclepro.2020.124863 (2021).

[3] Ogunkunle, O. & Ahmed, N. A. Overview of biodiesel combustion in mitigating the adverse impacts of engine emissions on the sustainable human-environment scenario. *Sustainability* **13**, 5465. 10.3390/su13105465 (2021).

[4] Al-Shetwi, A. Q. Sustainable development of renewable energy integrated power sector: Trends, environmental impacts, and recent challenges. *Sci. Total Environ.* **822**, 153645. 10.1016/j.scitotenv.2022.153645 (2022). 35124039 10.1016/j.scitotenv.2022.153645

[5] Bitire, S. O. & Jen, T.-C. Performance and emission analysis of a CI engine fueled with parsley biodiesel–diesel blend. *Mater. Renew. Sustain. Energy* **11**, 143–153. 10.1007/s40243-022-00213-4 (2022). 35892085 10.1007/s40243-022-00213-4 PMC9305677

[6] Neupane, D. et al. Growing Jatropha (*Jatropha curcas* L.) as a potential second-generation biodiesel feedstock. *Inventions* **6**, 60. 10.3390/inventions6040060 (2021).

[7] Balasubramanian, D. et al. Numerical and experimental evaluation on the pooled effect of waste cooking oil biodiesel/diesel blends and exhaust gas recirculation in a twin-cylinder diesel engine. *Fuel* **287**, 119815. 10.1016/j.fuel.2020.119815 (2021).

[8] Gharibian, M., Samani, B. H., Shirneshan, A. & Rostami, S. Investigation and ranking of the effect of biodiesel produced from safflower oil by the hydrodynamic method in diesel generator engine using TOPSIS method. *J. Renew. Energy Environ.* **9**, 13–22. 10.30501/jree.2021.272074.1186 (2022).

[9] Yesilyurt, M. K. The effects of the fuel injection pressure on the performance and emission characteristics of a diesel engine fuelled with waste cooking oil biodiesel-diesel blends. *Renew. Energy* **132**, 649–666. 10.1016/j.renene.2018.08.024 (2019).

[10] Bidir, M. G., Millerjothi, N. K., Adaramola, M. S. & Hagos, F. Y. The role of nanoparticles on biofuel production and as an additive in ternary blend fuelled diesel engine: A review. *Energy Rep.* **7**, 3614–3627. 10.1016/j.egyr.2021.05.084 (2021).

[11] Mahgoub, B. K. M. Effect of nano-biodiesel blends on CI engine performance, emissions and combustion characteristics—Review. *Heliyon* **9**, e21367. 10.1016/j.heliyon.2023.e21367 (2023). 38027745 10.1016/j.heliyon.2023.e21367 PMC10651469

[12] Gad, M. S. et al. A comprehensive review on the usage of the nano-sized particles along with diesel/biofuel blends and their impacts on engine behaviors. *Fuel* **339**, 127364. 10.1016/j.fuel.2022.127364 (2023).

[13] El-Seesy, A. I., Nour, M., Attia, A. M. A., He, Z. & Hassan, H. Investigation the effect of adding graphene oxide into diesel/higher alcohols blends on a diesel engine performance. *Int. J. Green Energy* **17**, 233–253. 10.1080/15435075.2020.1722132 (2020).

[14] Wang, S., Karthickeyan, V., Sivakumar, E. & Lakshmikandan, M. Experimental investigation on pumpkin seed oil methyl ester blend in diesel engine with various injection pressure, injection timing and compression ratio. *Fuel* **264**, 116868. 10.1016/j.fuel.2019.116868 (2020).

[15] Venu, H., Raju, V. D. & Subramani, L. Combined effect of influence of nano additives, combustion chamber geometry and injection timing in a DI diesel engine fuelled with ternary (diesel-biodiesel-ethanol) blends. *Energy* **174**, 386–406. 10.1016/j.energy.2019.02.163 (2019).

[16] Srinivasarao, M., Srinivasarao, Ch. & Kumari, A. S. Influence of Fe_3_O_4_ nanoparticles and compression ratio on the performance parameters of diesel engine using tamarind biodiesel: An experimental and ANN analysis. *Emiss. Control Sci. Technol.* **11**, 5. 10.1007/s40825-024-00255-2 (2025).

[17] Hasannuddin, A. K. et al. Nano-additives incorporated water in diesel emulsion fuel: Fuel properties, performance and emission characteristics assessment. *Energy Convers. Manag.* **169**, 291–314. 10.1016/j.enconman.2018.05.070 (2018).

[18] Okwu, M. O., Samuel, O. D., Ewim, D. R. E. & Huan, Z. Estimation of biogas yields produced from combination of waste by implementing response surface methodology (RSM) and adaptive neuro-fuzzy inference system (ANFIS). *Int. J. Energy Environ. Eng.* **12**, 353–363. 10.1007/s40095-021-00381-5 (2021).

[19] De Poures, M. V., Sathiyagnanam, A. P., Rana, D., Rajesh Kumar, B. & Saravanan, S. 1-Hexanol as a sustainable biofuel in DI diesel engines and its effect on combustion and emissions under the influence of injection timing and exhaust gas recirculation (EGR). *Appl. Therm. Eng.* **113**, 1505–1513. 10.1016/j.applthermaleng.2016.11.164 (2017).

[20] Fayad, M. A. et al. Reducing soot nanoparticles and NOX emissions in CRDI diesel engine by incorporating TiO_2_ nano-additives into biodiesel blends and using high rate of EGR. *Energies* **16**, 3921. 10.3390/en16093921 (2023).

[21] Ashok, B., Nanthagopal, K., Raj, R. T., Bhasker, J. P. & Vignesh, D. S. Influence of injection timing and exhaust gas recirculation of a *Calophyllum inophyllum* methyl ester fuelled CI engine. *Fuel Process. Technol.* **167**, 18–30. 10.1016/j.fuproc.2017.06.024 (2017).

[22] Nanthagopal, K. et al. A compressive review on the effects of alcohols and nanoparticles as an oxygenated enhancer in compression ignition engine. *Energy Convers. Manag.* **203**, 112244. 10.1016/j.enconman.2019.112244 (2020).

[23] Ramachandran, E. et al. Multicriteria decision-making technique for choosing the optimal ammonia energy share in an ammonia–biodiesel-fueled reactivity-controlled compression ignition engine. *ACS Omega* **9**, 5203–5214. 10.1021/acsomega.3c04005 (2024). 38343914 10.1021/acsomega.3c04005 PMC10851367

[24] Rao, P. M. et al. Artificial intelligence based modelling and hybrid optimization of linseed oil biodiesel with graphene nanoparticles to stringent biomedical safety and environmental standards. *Case Stud. Therm. Eng.* **51**, 103554. 10.1016/j.csite.2023.103554 (2023).

[25] Kari, J., Vanthala, V. S. P. & Sagari, J. Performance and emission characteristics of a diesel engine fuelled with *Mesua ferrea* biodiesel with chromium oxide (Cr_2_O_3_) nanoparticles: Experimental approach and response surface methodology. *Int. J. Thermofluids* **22**, 100637. 10.1016/j.ijft.2024.100637 (2024).

[26] Fu, J. et al. Application of artificial neural network to forecast engine performance and emissions of a spark ignition engine. *Appl. Therm. Eng.* **201**, 117749. 10.1016/j.applthermaleng.2021.117749 (2022).

[27] Sathyanarayanan, S. et al. Optimization of gasoline engine emission parameters employing commercial and sucrolite-catalyst coated converter using response surface methodology. *Int. J. Environ. Sci. Technol.* **20**, 1725–1738. 10.1007/s13762-022-03968-5 (2023).

[28] Uslu, S. Optimization of diesel engine operating parameters fueled with palm oil-diesel blend: Comparative evaluation between response surface methodology (RSM) and artificial neural network (ANN). *Fuel* **276**, 117990. 10.1016/j.fuel.2020.117990 (2020).

[29] Krishnamoorthi, M., Malayalamurthi, R. & Sakthivel, R. Optimization of compression ignition engine fueled with diesel—chaulmoogra oil—diethyl ether blend with engine parameters and exhaust gas recirculation. *Renew. Energy* **134**, 579–602. 10.1016/j.renene.2018.11.062 (2019).

[30] Vijay Kumar, M., Veeresh Babu, A. & Ravi, K. P. Experimental investigation on the effects of diesel and mahua biodiesel blended fuel in direct injection diesel engine modified by nozzle orifice diameters. *Renew. Energy* **119**, 388–399. 10.1016/j.renene.2017.12.007 (2018).

[31] Modi, V. et al. Nanoparticle-enhanced biodiesel blends: A comprehensive review on improving engine performance and emissions. *Mater. Sci. Energy Technol.* **7**, 257–273. 10.1016/j.mset.2024.02.001 (2024).

[32] Eslami, M. J., Hosseinzadeh Samani, B., Rostami, S., Ebrahimi, R. & Shirneshan, A. Investigating and optimizing the mixture of hydrogen-biodiesel and nano-additive on emissions of the engine equipped with exhaust gas recirculation. *Biofuels* **14**, 473–484. 10.1080/17597269.2022.2148877 (2023).

[33] Subramani, S., Govindasamy, R. & Rao, G. L. N. Predictive correlations for NOx and smoke emission of DI CI engine fuelled with diesel-biodiesel-higher alcohol blends-response surface methodology approach. *Fuel* **269**, 117304. 10.1016/j.fuel.2020.117304 (2020).

[34] Shirneshan, A., Kanberoglu, B. & Gonca, G. Experimental investigation and parametric modeling of the effect of alcohol addition on the performance and emissions characteristics of a diesel engine fueled with biodiesel-diesel-hydrogen fuel mixtures. *Fuel* **381**, 133489. 10.1016/j.fuel.2024.133489 (2025).

[35] Kumar, M., Kumar, V., Rajagopal, B. G., Samui, P. & Burman, A. State of art soft computing based simulation models for bearing capacity of pile foundation: A comparative study of hybrid ANNs and conventional models. *Model Earth Syst. Environ.* **9**, 2533–2551. 10.1007/s40808-022-01637-7 (2023).

[36] Moulali, P., Tarigonda, H. & Prasad, B. D. Optimization of performance characteristics of homogeneous charge compression ignition engine with biodiesel using artificial neural network (ANN) and response surface methodology (RSM). *J. Inst. Eng. India Ser. C* **103**, 875–888. 10.1007/s40032-021-00797-2 (2022).

[37] Youssef, A. & Ibrahim, A. A numerical investigation into the effect of altering compression ratio, injection timing, and injection duration on the performance of a diesel engine fuelled with diesel–biodiesel–butanol blend. *Clean Energy* **8**, 73–96. 10.1093/ce/zkae055 (2024).

[38] Rajesh Kumar, B., Saravanan, S. & Rajaram, K. Combined effect of oxygenates and injection timing for low emissions and high performance in a diesel engine using multi-response optimisation. *Alex. Eng. J.* **58**, 625–636. 10.1016/j.aej.2019.03.009 (2019).

[39] Duan, X., Xu, Z., Sun, X., Deng, B. & Liu, J. Effects of injection timing and EGR on combustion and emissions characteristics of the diesel engine fuelled with acetone–butanol–ethanol/diesel blend fuels. *Energy* **231**, 121069. 10.1016/j.energy.2021.121069 (2021).

[40] Dhahad, H. A., Fayad, M. A., Chaichan, M. T., Abdulhady Jaber, A. & Megaritis, T. Influence of fuel injection timing strategies on performance, combustion, emissions and particulate matter characteristics fueled with rapeseed methyl ester in modern diesel engine. *Fuel* **306**, 121589. 10.1016/j.fuel.2021.121589 (2021).

[41] Nayak, S. K. et al. Influence of injection timing on performance and combustion characteristics of compression ignition engine working on quaternary blends of diesel fuel, mixed biodiesel, and t-butyl peroxide. *J. Clean Prod.* **333**, 130160. 10.1016/j.jclepro.2021.130160 (2022).

[42] Santhosh, K. & Kumar, G. N. Effect of injection time on combustion, performance and emission characteristics of direct injection CI engine fuelled with equi-volume of 1-hexanol/diesel blends. *Energy* **214**, 118984. 10.1016/j.energy.2020.118984 (2021).

[43] Mobasheri, R., Aitouche, A., Pourtaghi Yousefdeh, S. & Zarenezhad, A. A. Assessing the impact of ethanol/biodiesel/diesel blends and nanoparticle fuel additives on performance and emissions in a DI diesel engine with EGR integration: An experimental study. *Processes* **11**, 1266. 10.3390/pr11041266 (2023).

[44] Soundararajan, G., Ponnusamy Kumarasami, D., Chidambaranathan, B. & Kasi, V. P. Influence of retarded injection timing on thermal performance and emission characteristics of a diesel engine fuelled with an optimized pyrolytic blend. *Energy Environ.* **33**, 1039–1060. 10.1177/0958305X211033970 (2022).

[45] Kalwar, A., Singh, A. P. & Agarwal, A. K. Experimental study of fuel injection timing and exhaust gas recirculation for combustion control in diethyl ether-diesel blend fuelled tractor engine. *Fuel* **371**, 131930. 10.1016/j.fuel.2024.131930 (2024).

[46] Devarajan, Y. Experimental evaluation of combustion, emission and performance of research diesel engine fuelled di-methyl-carbonate and biodiesel blends. *Atmos. Pollut. Res.* **10**, 795–801. 10.1016/j.apr.2018.12.007 (2019).

[47] Sengupta, A., Biswas, S. & Banerjee, R. Stratified injection strategies: Deciphering the emission and combustion synergies of n-butanol/diesel blends in single cylinder engines. *Fuel* **378**, 132939. 10.1016/j.fuel.2024.132939 (2024).

[48] Agarwal, A. K. et al. Dimethyl ether fuel injection system development for a compression ignition engine for increasing the thermal efficiency and reducing emissions. *Energy Convers. Manag.* **287**, 117067. 10.1016/j.enconman.2023.117067 (2023).

[49] Pv, E. et al. Split injection timing optimization in ammonia/biodiesel powered by RCCI engine. *Results Eng.* **23**, 102607. 10.1016/j.rineng.2024.102607 (2024).

[50] De Poures, M. V. et al. Collective influence and optimization of 1-hexanol, fuel injection timing, and EGR to control toxic emissions from a light-duty agricultural diesel engine fueled with diesel/waste cooking oil methyl ester blends. *Process Saf. Environ. Prot.* **172**, 738–752. 10.1016/j.psep.2023.02.054 (2023).

[51] Huang, H. et al. Effects of EGR rates on combustion and emission characteristics in a diesel engine with n-butanol/PODE3-4/diesel blends. *Appl. Therm. Eng.* **146**, 212–222. 10.1016/j.applthermaleng.2018.09.126 (2019).

[52] Yugandharsai, R., Jayaraman, J. & Reddy, S. Effects of injection pressure on performance & emission characteristics of CI engine using graphene oxide additive in bio-diesel blend. *Mater. Today Proc.* **44**, 3716–3722. 10.1016/j.matpr.2020.11.253 (2021).

[53] Yin, X. et al. Experimental analysis of the EGR rate and temperature impact on combustion and emissions characteristics in a heavy-duty NG engine. *Fuel* **310**, 122394. 10.1016/j.fuel.2021.122394 (2022).

[54] Ahmed, S. A. et al. Influence of injection timing on performance and exhaust emission of CI engine fuelled with butanol-diesel using a 1D GT-power model. *Processes* **7**, 299. 10.3390/pr7050299 (2019).

[55] Munuswamy, D. B., Devarajan, Y., Ramalingam, S., Subramani, S. & Munuswamy, N. B. Critical review on effects of alcohols and nanoadditives on performance and emission in low-temperature combustion engines: Advances and perspectives. *Energy Fuels* **36**, 7245–7268. 10.1021/acs.energyfuels.2c00930 (2022).

[56] Kumar, S. S. et al. Combustion, performance, and emission behaviors of biodiesel fueled diesel engine with the impact of alumina nanoparticle as an additive. *Sustainability* **13**, 12103. 10.3390/su132112103 (2021).

[57] Macián, V. et al. Cylinder-to-cylinder high-pressure exhaust gas recirculation dispersion effect on opacity and NO_*x*_ emissions in a diesel automotive engine. *Int. J. Engine Res.* **22**, 1154–1165. 10.1177/1468087419895401 (2021).

[58] Harun Kumar, M., Dhana Raju, V., Kishore, P. S. & Venu, H. Influence of injection timing on the performance, combustion and emission characteristics of diesel engine powered with tamarind seed biodiesel blend. *Int. J. Ambient Energy* **41**, 1007–1015. 10.1080/01430750.2018.1501741 (2020).

[59] Raju, V. D. et al. Comprehensive analysis of compression ratio, exhaust gas recirculation, and pilot fuel injection in a diesel engine fuelled with tamarind biodiesel. *Sustainability* **15**, 15222. 10.3390/su152115222 (2023).

[60] Jayabal, R., Thangavelu, L. & Subramani, S. Combined effect of oxygenated additives, injection timing and EGR on combustion, performance and emission characteristics of a CRDi diesel engine powered by sapota biodiesel/diesel blends. *Fuel* **276**, 118020. 10.1016/j.fuel.2020.118020 (2020).

[61] Bello, U. et al. Enhancing oxidative stability of biodiesel using fruit peel waste extracts blend: Comparison of predictive modelling via RSM and ANN techniques. *Results Eng.* **21**, 101853. 10.1016/j.rineng.2024.101853 (2024).

